# Algorithms and Methods for Individual Source Camera Identification: A Survey

**DOI:** 10.3390/s25103027

**Published:** 2025-05-11

**Authors:** Jaroslaw Bernacki, Rafal Scherer

**Affiliations:** 1 Department of Artificial Intelligence, Czȩstochowa University of Technology, al. Armii Krajowej 36, 42-200 Czȩstochowa, Poland; 2 Faculty of Computer Science, AGH University of Krakow, 30-059 Kraków, Poland

**Keywords:** digital forensics, digital camera identification, machine learning, deep models, privacy

## Abstract

Source camera identification (SCI) is a key issue in the field of digital forensics. This paper presents a comprehensive review of the existing methods and algorithms used for this purpose. It discusses approaches based on matrix noise analysis, including methods utilizing sensor pattern noise, photo response non-uniformity, statistical methods, aberrations analysis, as well as modern techniques based on deep neural networks and machine learning. Particular attention is paid to the effectiveness and robustness of the algorithms to different types of interference and their possible application in practical cases, such as law enforcement investigations. Moreover, we also discuss the issue of camera identification using videos and provide a brief description of popular image datasets that might be used for source camera identification method benchmarking.

## 1. Introduction

The development of digital technology and the vast use of cameras in everyday life make the issue of identifying the source of images important. Identifying the camera or sensor that took a specific image not only supports law enforcement activities but also plays a significant role in the analysis of photo authenticity and research on image analysis and digital forensics. Sensor identification analysis based on images (often understood as source camera identification—SCI) plays a key role in the context of privacy protection, especially in the era of increasing digitalization and easy access to tools for editing and publishing images. Modern technologies enable not only the creation of high-quality images but also their easy modification, which, combined with their widespread sharing on the network, poses serious threats to the privacy of individuals.

The basis of many source camera identification (SCI) methods is the analysis of unique noise characteristics generated by image matrices, which is generally called sensor pattern noise (SPN). Each digital sensor, regardless of manufacturer, introduces specific distortions into the image, which are a result of its design and manufacturing process. These features, often referred to as the camera fingerprint, can be analyzed to assign an image to a specific camera model [1,2].

The SCI is typically realized in two aspects: individual source camera identification (ISCI) and source camera model identification (SCMI). The difference between ISCI and SCMI is crucial in the context of digital forensics image analysis. Individual source camera identification involves determining which specific camera was used to capture a given image, which often relies on unique characteristics such as sensor noise resulting from manufacturing imperfections [2,3]. This is a more complex process because it requires referencing a database specific to each device. In contrast, the SCMI focuses on classifying images into a specific group of camera models, which is possible by analyzing common characteristics of a given production line, such as lens parameters or image processing algorithms. This form of identification is usually less precise but much faster because it requires less comparative data. The SCMI aspect is usually used for fast image pre-processing [4]. Both approaches are important in the context of digital forensics, but their applications and goals differ depending on the needs and context of the analysis [5]. In the majority of cases, research papers deal with the ISCI aspect. In this paper, we will understand the SCI as the ISCI aspect.

**Figure 1:**
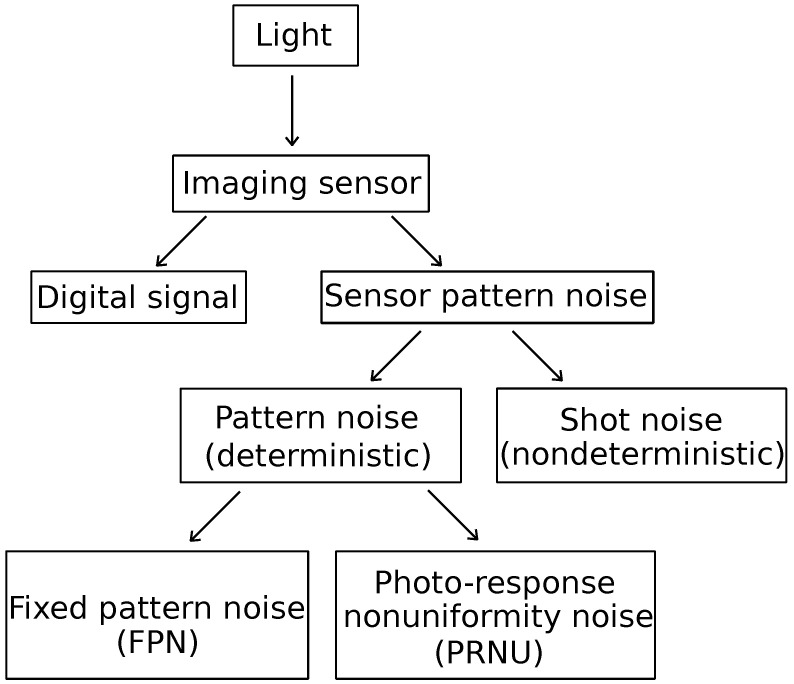
Classification of noise occurring in imaging sensors. The noise generated by the sensor consists of deterministic pattern noise and nondeterministic shot noise. The pattern noise is made up of fixed pattern noise and photo response non-uniformity noise [6].

One of the key issues in identifying devices is SPN analysis. Its main components include the deterministic pattern noise and the random noise (also known as shot noise). The pattern noise consists of fixed pattern noise (FPN) and photo response non-uniformity (PRNU). FPN is caused by dark currents and it primarily refers to the differences between pixels when the imaging sensor is not exposed to light. Because FPN is additive noise, some mid- to high-end consumer digital cameras automatically suppress this noise by subtracting a dark frame from each image taken. FPN is also dependent on exposure and temperature. In natural images, the dominant part of sensor pattern noise is PRNU which is caused by pixel non-uniformity. This is defined as the different sensitivity of pixels to light and is caused by silicon wafer inhomogeneity and imperfections during the imaging sensor manufacturing process [2,6]. PRNU serves as a unique fingerprint for each camera and can be derived from multiple images. The classification of the sensor noise is presented in Figure 1.

One of the most important works on the subject of SCI is the algorithm presented by Luk’as et al. [2]. This paper introduces a method for the ISCI aspect through the analysis of photo response non-uniformity. The approach involves determining a reference pattern noise for each camera, which acts as a unique fingerprint, by averaging noise from multiple images using a denoising filter. For identification, this reference pattern noise is treated as a spread-spectrum watermark, detected in the image using a correlation method. The authors conduct experiments using images coming from several consumer digital cameras to estimate false alarm and false rejection rates, examining how these error rates are affected by common image processing techniques, including JPEG compression and gamma correction. The main assumptions of the algorithm can be formalized as follows. For a given image I, the noise residual W is calculated by filtering out the image structures. Typically, wavelet-based or low-pass filters are used, which remove the main features of the image, leaving the noise residual in the following manner:

(1) W=I−F(I)

where *F* is a denoising filter. The PRNU pattern K for a given camera is estimated by averaging the noise residuals from multiple images taken with the same camera:

(2) $$K=\frac{1}{N}\sum_{i=1}^{N}W_{i}$$

where *N* is the number of images, and $W_{i}$ is the noise residual from the *i*-th image. To identify whether a given image I comes from a particular camera, its noise residual $W_{i}$ is compared to the stored PRNU pattern K using a correlation technique.

Bondi et al. [7] proposed a method for source camera identification using a convolutional neural network (CNN) trained on SPN features. This approach partially builds on Luk’as’ earlier work but modernizes it by utilizing deep learning techniques for improved performance. The CNN consisted of several convolutional layers designed to learn spatial hierarchies of image features related to the sensor pattern noise and is trained with the noise residuals calculated in the same manner, as shown in Equation (1). The network also included pooling layers to reduce spatial dimensions and emphasize the most relevant features, followed by two fully connected layers that performed the final classification. The authors demonstrated that this CNN-based method outperformed traditional SPN extraction techniques, such as those based on statistical analysis, achieving higher accuracy in camera source identification.

In this survey, we present a comprehensive overview of the methods used for source camera identification (SCI), particularly in the individual source camera identification (ISCI) aspect, focusing on both traditional techniques and modern deep learning-based approaches. The classical methods, such as sensor pattern noise (SPN), photo response non-uniformity (PRNU), aberration analysis, and statistical models, form the foundational techniques for source camera identification. However, advancements in deep learning, including convolutional neural networks (CNNs) have enhanced these approaches, utilizing them to improve the accuracy of identification methods. This work explores both the core identification techniques as well as the modern algorithms that apply and extend them.

### Organization of the Paper

This paper is organized as follows. The next section mentions the image datasets that might be used to test the methods for source camera identification. In Section 3, the identification using sensor pattern noise is described. In Section 4, the photo response non-uniformity-based methods are mentioned. The next section discusses the deep model methods. Section 6 and Section 7 depict identification methods utilizing aberrations analysis and statistical methods, respectively. Section 8 discusses the robustness and adversarial attacks on the camera’s fingerprint. The next sections describe the identification using videos and other methods. Finally, Section 11 summarizes the paper, with the directions of open issues and future work. At the end of the survey, there is an Abbreviations section, which contains the list of all abbreviations and their corresponding full names of the methods cited in the paper. Throughout the paper, in the formal descriptions, the bold font denotes matrices or vectors.

## 2. Image Datasets

Testing source camera identification algorithms requires large and diverse datasets that accurately represent real-world conditions. Such datasets should provide the necessary data for evaluating the performance of algorithms, allowing for the analysis of accuracy, robustness, and potential vulnerabilities under various scenarios. In this section, we discuss the importance of the most frequently used datasets in algorithm testing, underlining their role in ensuring the reliability of identification methods.

One of the most important image datasets for SCI is the Dresden Image Database [8]. It is a comprehensive dataset of over 14,500 images designed for developing and benchmarking camera-based digital forensic techniques. Acquired under controlled indoor and outdoor conditions, the images were taken with 73 cameras across 25 different models, allowing the distinguishing of device-specific and model-specific characteristics. Additionally, images for estimating sensor noise and model-specific JPEG compression samples were included.

The article by De et al. [9] introduced MICHE-I, an iris biometric dataset captured under uncontrolled conditions using various mobile devices. Key features include a diverse subject population, multiple devices, simulated acquisition noise, multiple data capture sessions, and metadata annotations. The dataset addresses critical biometric challenges such as uncontrolled settings, demographic diversity, and real-world applications, including continuous authentication to counter spoofing and impersonation in different scenarios. Although this dataset is mainly dedicated to iris research, it may be used for source camera identification tasks, since it includes iris images captured by different mobile devices. The number of images is 3700 from 92 mobile devices.

In the work by Shullani et al. [10], the VISION dataset is introduced. It is developed to support multimedia forensics research in a realistic context where portable devices and social media significantly impact data characteristics. With 34,427 images and 1914 videos from 35 devices across 11 brands, the dataset includes both original and social media-compressed versions (from platforms like Facebook, YouTube, and WhatsApp). VISION addresses challenges like digital stabilization, which is common in modern cameras, and social media re-compression, which can affect forensic algorithms’ reliability. By capturing both images and videos with the same sensor, VISION offers a benchmark for evaluating forensic tools on realistic, device-specific, and content-shared data.

The Forchheim Image Database (FODB), designed for SCI and image provenance tracking, was presented by Hadwiger et al. [11]. The FODB consists of over 23,000 images across 143 distinct scenes, captured by 27 smartphones. It uniquely separates image content from forensic artifacts, offering each image in six quality levels: the original version and five versions with social media recompression. Therefore, this structure supports benchmarking under realistic conditions, as demonstrated in tests where general-purpose EfficientNet outperformed forensic-specific CNNs.

In [12], the SCI in digital forensics, focusing on the High Dynamic Range (HDR) images compared to Standard Dynamic Range (SDR) images is addressed. The authors introduce a database called UNIFI, containing HDR and SDR images captured under varied conditions, including different motions, scenes, and devices. Testing a reference pattern noise-based algorithm revealed that HDR images pose greater difficulties for source identification, likely due to their complexity and expanded dynamic range. The findings suggest that both capturing conditions and device variations significantly influence identification performance, highlighting areas for further research in HDR image forensics.

The IMAGIng seNsor idEntification (IMAGINE) dataset was introduced in 2023 by Bernacki & Scherer as a new resource for benchmarking camera identification algorithms in digital forensics [13]. Unlike the widely used Dresden Image Database, which mainly consists of images from older devices using CCD sensors, the IMAGINE dataset features images from modern devices, including mobile phones, compact cameras, and digital single lens reflex/mirorless cameras equipped with CMOS sensors. It contains 2816 images coming from 67 devices, also from several copies of the same model. The dataset aims to provide more relevant testing conditions for forensic methods, as confirmed by extensive experimental evaluations that highlight its reliability. IMAGINE supports the development and assessment of algorithms for identifying image sources with current digital imaging technology.

In the work by Akbari et al. [14], a video database called Qatar University Forensic Video Database (QUFVD) is presented. It contains 6000 videos recorded on 20 devices of different brands, including identical models. Initial tests of camera identification using deep learning indicate difficulties in accurate device identification, emphasizing the need for further research in this area.

The paper by Galdi et al. [15] presents the SOCRatES database (SOurce Camera REcognition on Smartphones) that comprises approximately 9700 images and 1000 videos captured with 103 smartphones from 15 different brands and around 60 distinct models. All images and videos are taken in uncontrolled conditions. This approach was designed to gather a heterogeneous dataset, maximizing the variety of devices and simulating real-world scenarios where this database will serve as a benchmark. The database covers a broad range of commercially available devices.

The Daxing dataset [16] was specifically developed to support research in the source identification of images and videos captured with smartphones. Recognizing the shift from traditional digital cameras to ubiquitous smartphone usage, this dataset reflects the modern landscape of digital media acquisition, where nearly every individual owns a smartphone capable of producing vast amounts of visual content. Daxing contains a total of 43,400 images and 1400 videos, collected from 90 smartphones spanning 22 models across five major brands. Notably, it includes multiple instances of the same device model, for instance, 23 different iPhone 6S (Plus) units, enabling robust evaluation of inter-device variability. Compared to other available datasets, Daxing offers one of the largest and most diverse collections in terms of device count, per-model coverage, and the volume of media. This makes it particularly valuable for forensic and scientific studies on device-level source attribution. The dataset is publicly available and free for research and investigative purposes.

Some social media like Flickr [17] can also be a valuable source of images for camera identification research. The Flickr platform does not modify uploaded images itself, preserving their original features. However, caution should be taken as users may make modifications before uploading, which can compromise the authenticity of the image data. In such cases, it is important to verify that the images have not been edited to ensure the reliability of the research results.

All datasets considered in this study primarily consist of RGB images in JPEG format, which is the standard output format of most consumer digital cameras and mobile devices, particularly the following:

- Dresden Image Database, Forchheim Image Database, UNIFI, and IMAGINE datasets contain RGB images in JPEG format, with typical resolutions ranging from 1024×768;
- MICHE-I includes RGB iris images captured with mobile devices, also in JPEG format.
- VISION and SOCRatES provide both images and videos. The image components are JPEGs, while videos are typically stored in .mp4 containers with resolutions up to Full HD;
- QUFVD consists exclusively of videos, which are provided in the .mp4 format and contain RGB content at common consumer camera resolutions;
- Daxing dataset includes 43,400 JPEG images and 1400 MP4 videos captured using 90 smartphones across 22 models and 5 brands.

Due to the large number and diversity of images in the surveyed datasets, it is not feasible to provide a comprehensive list of their resolutions. Most datasets contain images captured with various consumer devices, often including multiple resolutions even within the same collection. The video resolutions range from VGA up to Full HD and higher, depending on the recording device and acquisition settings.

A summary of the datasets for the SCI is presented in Table 1.

## 3. Identification by Sensor Pattern Noise (SPN)

Sensor pattern noise (SPN) is a unique, device-specific noise pattern present in digital images due to imperfections in camera sensor manufacturing. Unlike random noise, SPN remains consistent across all images taken by the same sensor, making it a powerful tool for SCI. This section explores the application of SPN in linking digital images to their originating devices with the distinction between traditional and deep learning-based methods.

In the paper proposed by Kang et al. [18], a method for SCI using SPN as a unique fingerprint is presented. The approach addresses challenges posed by image content, JPEG compression artifacts, and other factors that can contaminate SPN. By modeling SPN as additive white Gaussian noise, the authors propose a two-step process: first, whitening the noise residues extracted from original images, then averaging them to create a reference SPN. Utilizing a correlation to circular correlation norm (CCN), the method effectively reduces the false positive rate compared to traditional peak-to-correlation energy (PCE) methods. Theoretical analysis indicates that the proposed method enhances SCI performance. Experimental results across seven cameras, using a total number of 1400 images, demonstrate that the proposed method outperforms existing SCI techniques, particularly in its resistance to JPEG compression, achieving much better receiver operating characteristic (ROC) performance in all tested scenarios. In the study [19], an SCI method utilizing SPN as a device fingerprint for image origin verification is discussed. Recognizing that SPN can be significantly contaminated by image content, particularly in the presence of strong scene details like edges, the authors propose an SPN predictor based on an eight-neighbor context-adaptive interpolation algorithm to soften these effects. This approach enhances the purity of the estimated SPN, leading to improved performance for SCI. Experimental results across various image datasets demonstrate that the proposed method overtakes existing SCI techniques, especially in resisting mild JPEG compression while maintaining low false-positive rates crucial for reliable SCI in practical applications. However, the method requires a substantial number of original images (at least 100) to establish an effective camera fingerprint, and its efficacy is much lower when fewer images are available.

Another framework for SCI utilizing SPN is presented by Li et al. in [20]. Because of the high dimensionality and computational costs associated with SPN extraction from large image blocks, the authors utilize principal component analysis (PCA) for denoising in the SCI task. The proposed framework formulates a compact SPN representation while minimizing the impact of interfering artifacts through a specialized training set construction. The method is effective even when only textured reference images are available. To further enhance identification performance, linear discriminant analysis (LDA) is employed to extract more discriminative SPN features. Extensive experiments conducted on the Dresden Image Database demonstrate that this approach not only improves the SCI performance but also significantly reduces computational costs during the matching phase, making it an effective post-processing solution for practical applications.

The challenge of accurately identifying source cameras using SPN by using a method to create a reliability map for SPN extraction is described in the work by Satta et al. [21]. High-frequency components, such as textures and edges, can be mistaken for SPN due to the adaptive low-pass filtering used in the extraction process. To lower this effect, the proposed method evaluates the reliability of each pixel based on the high-frequency content in its neighborhood. This reliability map is then utilized to weigh SPN pixels during the matching process. Testing on a dataset of images from 27 different cameras demonstrated significant improvements in identification accuracy compared to traditional non-weighted matching techniques.

The work proposed by Soobhany et al. [22] presents a wavelet-based method for SCI using SPN. The approach uses a non-decimated wavelet transform to decompose images into high-frequency sub-bands, from which the SPN is extracted. Cross-correlation between the image SPN and a reference SPN signature allows for accurate source identification. Tested on images from ten cameras, the method demonstrates improved performance over existing wavelet-based techniques, offering higher accuracy in identifying the cameras.

Zeng et al. [23] proposed a method for extracting the SPN using dual-tree complex wavelet transform (DTCWT) to improve the quality of SPN. Traditional methods, such as discrete wavelet transform (DWT), struggle with edge areas and image borders, leading to poor SPN quality. The proposed DTCWT method enhances SPN extraction near strong edges and employs symmetric boundary extension to improve results along the image border. Extensive experiments on a representative number of images demonstrate that this method outperforms existing techniques in SCI and shows promising results for image tampering localization.

The study presented by Kulkarni et al. [24] focused on sensor imperfections by utilizing the SPN for identification across various imaging sensors. The proposed system introduces a method for extracting sensor noise from database images using gradient-based and Laplacian operators, creating a hybrid approach that highlights edges and noise. After removing edges, the resulting noisy image is analyzed using the gray-level co-occurrence matrix (GLCM) to extract features such as homogeneity, contrast, correlation, and entropy. These features are then used to evaluate performance and matching accuracy against the test set, demonstrating that the combination of SPN extraction and GLCM feature analysis significantly enhances identification results.

In the study by Gupta et al. [25], a method of enhancing the SCI by preprocessing SPN is described. The authors highlight that low-frequency artifacts, often caused by light refraction on dust and optical surfaces, can increase the false acceptance rate in camera recognition. Therefore, a method that combines spectrum-equalization algorithms (SEAs) to suppress peaks in the SPN with techniques to eliminate low-frequency defects in the discrete cosine transform domain is proposed. Experimental results on the Dresden Image Database demonstrate that this approach significantly improves the efficacy of existing SEA methods and outperforms other SPN enhancement techniques, leading to better accuracy and reliability in identifying digital cameras.

A framework to enhance SCI by improving the extraction of SPN from a single test image is discussed by Kirchner & Johnson [26]. Unlike traditional methods that use denoising filters, which often overlook the specific SPN signal, it is proposed that a deep learning approach serves as a more effective extractor for improved source attribution. Extensive experiments conducted on multiple datasets validate the efficacy of the proposed method, demonstrating its applicability not only in the SCI but also in image manipulation localization and video source attribution.

Qian et al. [27] depicted a neural network utilizing SPN. The framework involves three key stages: registering the device fingerprint, extracting the fingerprint during photo capture, and verifying the connection between photos and their source devices. By integrating metric learning and frequency consistency into the deep network design, the proposed fingerprint extraction algorithm achieves state-of-the-art performance with modern smartphone images. Moreover, two cryptographic schemes, fuzzy extractor and zero-knowledge proof (ZKP), are also introduced for reliable correlation between registered fingerprints and verified photo fingerprints.

The work proposed by Mandelli et al. [28] addressed a fast and accurate SCI method based on SPN using CNNs. Specifically, it utilizes a two-channel CNN that compares camera fingerprints with image noise at the patch level. This approach significantly enhances speed and accuracy compared to conventional methods, making it particularly effective for analyzing large image databases. Additionally, the study investigates the impact of double JPEG compression on images, also reporting higher accuracy than standard approaches. A deep learning approach for managing the SPN is also presented in [29]. A summary of the SPN-based SCI methods is presented in Table 2.

While Table 2 summarizes various SPN-based source camera identification methods, a direct performance comparison between traditional and deep learning-based techniques remains difficult due to the use of different datasets, preprocessing steps, and evaluation protocols across the literature. However, recent studies suggest that deep learning methods tend to outperform traditional approaches, especially in scenarios involving compression, resizing, or real-world noise [7,30]. Nonetheless, traditional techniques remain relevant due to their interpretability, lower computational cost, and robustness in controlled settings.

### Discussion

Sensor pattern noise has long been regarded as one of the most reliable and device-specific features for source camera identification (SCI). The reviewed methods confirm the strength of SPN as a unique fingerprint, particularly in scenarios involving multiple devices of the same model. This characteristic makes SPN invaluable in digital forensics, where device-level discrimination is often required.

A consistent trend observed across the reviewed studies is the effort to suppress or lower the interference from image content, such as scene texture and edges. Since SPN is embedded beneath image structures, these elements can significantly contaminate the noise residual, leading to reduced identification accuracy. Approaches such as context-adaptive interpolation [19] and SPN whitening [18] highlight the importance of preprocessing techniques that enhance the purity of extracted SPN. The integration of statistical models (e.g., additive white Gaussian noise) and advanced denoising strategies (e.g., PCA [20]) further reflects a growing sophistication in SPN modeling and extraction.

Despite these improvements, several practical limitations remain. Many SPN-based methods require a large number of reference images to construct a stable device fingerprint. This requirement limits the applicability of SPN techniques in real-world cases where access to original images may be restricted. Furthermore, SPN extraction is computationally intensive, especially when high-resolution images are involved. Methods that utilize dimensionality reduction, such as PCA and LDA, offer promising directions to optimize the trade-off between accuracy and efficiency, but further work is needed to make SPN-based SCI scalable for large datasets and real-time analysis.

Although numerous SPN-based methods have been proposed and evaluated in the literature, direct comparisons across datasets remain limited. Most works report results on different datasets, under varying experimental setups and evaluation protocols, which makes it difficult to assess their relative performance objectively. To the best of our knowledge, only a few studies attempt to benchmark multiple approaches under unified conditions, and even these differ in preprocessing steps, network architectures, or evaluation metrics. This highlights a significant gap in the field and suggests a need for standardized benchmarks and shared evaluation protocols for fair comparison of SPN-based techniques. These observations highlight the need for future benchmark studies that systematically compare SPN-based techniques under unified settings, taking into account cross-dataset and cross-device generalization performance.

Another important challenge concerns robustness against post-processing operations. While recent methods show improved resistance to JPEG compression and scene interference, robustness under more aggressive conditions (e.g., resizing, filtering, or malicious noise injection) is still a topic of active research. Additionally, the integration of SPN with modern deep learning approaches, either as a preprocessed input or a regularization signal, has not been fully explored, representing a potentially fruitful area for hybrid methodologies.

In conclusion, SPN remains a cornerstone technique in SCI, combining strong theoretical foundations with demonstrable forensic utility. To further enhance its relevance and applicability, future research should focus on reducing the data dependency of SPN fingerprinting, improving robustness to image manipulation, and investigating synergy with learning-based frameworks.

## 4. Identification by Photo Response Non-Uniformity (PRNU)

Photo response non-uniformity (PRNU) is a distinctive pattern of pixel-level variations in light sensitivity across a digital camera sensor, coming from manufacturing imperfections. This unique, stable pattern acts as a “fingerprint” for each camera, allowing images to be traced back to the specific device that captured them. In this section, we examine PRNU characteristics, techniques for extracting it from images, and its application in identifying source cameras.

A method for grouping images from a database based on PRNU patterns is described in Baar et al. [31]. By extracting these noise patterns with optimized filters, the method significantly reduces computation time compared to other approaches. Images with noise pattern correlations above a set threshold can be quickly matched, enabling the identification of groups of images taken by the same camera from a large database. This approach is effective for scanning extensive image collections for suspect noise patterns.

The work by Filler et al. [1] depicts the application of PRNU as a reliable method for the SCI. The authors demonstrate that this fingerprint generated from TIFF and JPEG images can be utilized to identify the camera’s brand and model. By extracting a set of numerical features and employing pattern classification techniques, the study evaluates fingerprints from over 4500 digital cameras across eight brands and 17 models. The results indicate an average classification accuracy of 90.8% for identifying the correct camera brand.

The exploration of digital imaging sensor imperfections as unique identifiers for various forensic applications is discussed in the work by Fridrich et al. [32]. The sensor imperfections can aid in matching images to specific cameras, detecting malicious image manipulation, and estimating the age of digital photographs. The authors categorize different types of defects resulting from manufacturing flaws, internal camera processes, and environmental factors, emphasizing their forensic relevance. The study also formulates specific detection and matching tasks related to these defects and noise patterns. It develops practical algorithms within the context of parameter estimation and signal detection theory, demonstrating their effectiveness.

The paper proposed by Gisolf et al. [33] proposes a method for extracting the PRNU noise. Traditional methods, particularly wavelet-based denoising, can be time-consuming, especially when dealing with a large number of images. The proposed approach enhances the speed of PRNU extraction while maintaining accuracy, utilizing a simplified version of the total variation noise removal algorithm. Experimental results demonstrate that this method is approximately 3.5 times faster than the wavelet-based standard. Moreover, it outperforms the existing methods in terms of accuracy, suggesting that the total variation approach offers both efficiency and improved performance for the SCI tasks.

The work by Gupta et al. [34] underlines that sensor imperfections like PRNU are valuable not only for image forensic tasks like SCI but also image integrity verification and device linking. However, existing PRNU extraction methods often retain high-frequency details (edges and textures) from the image, which can obscure the weak noise signal that PRNU represents. Therefore, a preprocessing step to enhance commonly accepted PRNU extraction methods is introduced. The approach utilizes the principle that PRNU can be effectively extracted by applying the extraction methods to low-frequency (LF) and high-frequency (HF) components of the image. Initial experiments with this preprocessing concept demonstrated significant performance improvements across most PRNU extraction methods, with the Mihcak filter showing the most substantial enhancement.

The paper proposed by Darvish et al. [35] focuses on the issue of geometric operations applied to images during their acquisition and processing. These operations, such as rotation, downsampling, and cropping, can lead to pixel desynchronization, which results in complicating the identification process. The goal of this work is to enhance the applicability of PRNU-based camera identification methods to high dynamic range (HDR) images. For this purpose, an approach that addresses the geometric transformations present in HDR images is proposed. The method involves a series of steps to reverse these transformations through block-wise PRNU matching. Such a strategy allows for effectively realigning the PRNU signals and maintaining their integrity despite the alterations imposed by geometric operations. As an evaluation, the experiments using HDR images from the UNIFI dataset from 26 different mobile devices are conducted. The results demonstrate that the proposed method significantly improves the performance of the SCI in the context of HDR images, providing a robust solution to the pixel desynchronization challenge.

The identification of iris sensors used to capture iris images through the PRNU noise analysis is investigated in Kalka et al. [36]. By generating a noise reference pattern for each sensor, the study correlates these patterns with noise residuals from iris images across several databases. The results are promising, showing identification rates between 86.0% and 99.0% for unit-level testing (same vendor) and 81.0% to 96.0% for brand-level testing (different vendors). The findings indicate that sensor identification is feasible even with a limited number of training images.

In the work by Marra et al. [37], an algorithm for blind camera identification utilizing PRNU noise estimated from image residuals is presented. The identification process relies on residuals originating from the same camera. The approach follows a two-step strategy: first, it employs correlation clustering to efficiently group residuals, intentionally over-partitioning the data to prevent incorrect associations. In the second step, basic clusters are merged using a specialized refinement algorithm. Experiments conducted on the Dresden Image Database demonstrate the efficacy of the proposed method in achieving reliable camera identification.

The paper proposed by Tiwari et al. [38] addresses the challenges in SCI using PRNU. The standard PRNU extraction method, which involves applying a denoising filter and calculating the difference between the original and denoised images, can be adversely affected by intensity-based features and high-frequency details (like edges and textures). To address these issues, the authors propose a weighting function that considers these image features. By experimentally identifying the impact of intensity and high-frequency content on the estimated PRNU, they develop a function that assigns higher weights to regions providing reliable PRNU while reducing weights for less reliable areas. Experimental results demonstrate that this weighting function significantly enhances the accuracy of SCI.

In the work by Tomioka et al. [39], an enhanced method for SCI is introduced. The proposed approach clusters PRNU noises to extract sensor features that are robust to random noise, scene content, and image processing effects like noise reduction. Experimental results demonstrate that this method achieves high identification accuracy, which confirms its efficacy.

The work proposed by Valsesia et al. [40] explores the use of compressed PRNU patterns to the SCI. The authors propose using random projections to compress the PRNUs while maintaining matching accuracy. The performance of these randomly projected fingerprints is analyzed theoretically and validated through experiments on real image databases. Additionally, the paper discusses practical considerations for implementing random projections, including the use of circulant matrices.

The research in the work by Bernacki [41] proposes an algorithm that significantly accelerates the SCI compared to state-of-the-art methods. The method utilizes an algebraic operation applied to the input images in order to obtain their fingerprints in compressed representations. Experimental evaluation on two large datasets, comprising nearly 14,000 images, demonstrate that the proposed algorithm achieves a high classification accuracy of 97.0%, outperforming existing methods that yield 92.0–96.0%. Additionally, a statistical analysis of the results supports their reliability.

The paper by Manisha et al. [42] discusses the limitations of PRNU noise patterns in SCI, particularly their vulnerability to camera settings, image processing, and counter-forensic attacks. The authors introduce a robust data-driven device-specific fingerprint that can effectively identify individual cameras of the same model. Unlike PRNU, this new fingerprint is location-independent, stochastic, and globally available, which resolves the spatial synchronization issue associated with PRNU matching. The proposed fingerprint is extracted from the low- and mid-frequency bands of digital images, making it more resilient to common image manipulations, such as rotation, gamma correction, and aggressive JPEG compression. Experimental results across various datasets indicate that such fingerprint significantly enhances the ability to identify source cameras in practical forensic scenarios.

The work proposed by Taspinar et al. [43] demonstrates that by using seam-carved images containing uncarved blocks below 50×50 pixels, the source identification remains feasible. By theoretical and experimental analysis, the study finds that the success of attribution depends on both the number of seams removed and the randomness of their placement, providing suggestions for improving PRNU resilience against such countermeasures.

The paper described by Rodriguez et al. [44] deals with the issue of SCI, focusing on the challenge of identifying cameras using natural images instead of flat images. The proposed approach utilizes a statistical comparison of the noise residual in naturally disputed images against the PRNUs from various digital cameras. The method employs the HDR image database to generate the PRNU fingerprints from the probability density function (PDF), extracted from reference images. To identify the source camera of a natural disputed image, the authors implement Jensen–Shannon divergence (JSD) to statistically compare the PRNU-based fingerprints of candidate cameras with the noise residual of the disputed image. The results indicate that this method achieves effectiveness comparable to peak-to-correlation energy and Kullback–Leibler divergence methods for flat images but significantly outperforms them when applied to natural images.

In the paper proposed by Lawgaly et al. [45], the challenges posed by image-dependent information and non-unique noise components during the PRNU extraction process are addressed. The authors propose enhancements across three stages: filtering, estimation, and post-estimation. An improved locally adaptive discrete cosine transform filter is introduced for better noise reduction in the filtering stage, while a weighted averaging technique is employed in the estimation stage. The post-estimation stage involves concatenating PRNUs from different color planes to utilize the physical PRNU components. Experimental results on datasets from various camera devices demonstrate significant performance improvements at each stage and highlight the overall efficacy of the proposed system compared to existing state-of-the-art methods.

In the work proposed by Chen et al. [46], the investigation of the effectiveness of PRNU-based image tampering detection methods is presented. The experiments are conducted with PRNU forensic techniques to evaluate their performance on images captured by various digital single-lens reflex cameras. Over 800 PRNU noise patterns were generated from 80 photos to assess the similarities between intra-class and inter-class PRNU patterns. The analysis highlights the performance of existing methods and offers suggestions for future research and development in PRNU-based image forensics.

The work described by Yaqub et al. [47] addresses the challenge of using PRNU-based camera fingerprints for identifying the source camera of anonymous images, particularly focusing on the high run time overhead associated with large camera databases. While existing techniques aim to reduce computation time through fingerprint size reduction, they often fail when images are scaled or cropped. The authors propose a scaling-based approach that maintains effective SCI for full-resolution or cropped images. This method can be seamlessly integrated with existing PRNU matching techniques for scaled images. Experiments involving 250 cameras demonstrate that the proposed approach can reduce run time when processing cropped images.

The paper by Goljan et al. [3] reviews the state-of-the-art methods using the PRNU. Traditional methods, which test multiple camera fingerprints against individual images, can be time-consuming and often yield unreliable results in tail extrapolation of correlation distributions. The authors propose an approach utilizing cross-correlation analysis and the peak-to-correlation energy ratio to enhance efficiency and reliability in the camera identification process.

In the work described by Borole et al. [48], a method that utilizes gray-level co-occurrence matrix (GLCM) is presented. The PRNU noise is extracted from denoised images using a denoising filter, and the GLCM features of the PRNU noise pattern are then computed. For classification, the framework employs a scaled conjugate gradient (SCG) backpropagation neural network, aiming to enhance the accuracy of camera identification. The paper [49] presents a feature-based approach where the input image is denoised to extract the PRNU noise pattern. These patterns are represented using Hu’s invariants, which remain consistent under scaling, translation, and rotation. The extracted features are then utilized to train and classify using a fuzzy min-max neural network (FMNN). The proposed method demonstrates the capability to identify cameras that capture the same scene, enhancing the effectiveness of the SCI.

In the work presented by Ahmed et al. [50], a comparison of traditional filtering-based techniques with CNNs is investigated. The study focuses on the basic sensor pattern noise estimation using the Wiener filter in the wavelet domain and evaluates its efficacy against a deep learning CNN model. Experiments were conducted on a dataset comprising images from eleven different cameras, utilizing consistent training and test images of size 128×128 and 256×256 pixels for both methods. The findings reveal that the filtering-based PRNU approach for SCI demonstrates much better performance in terms of overall accuracy, false positive rates, and false negative rates, particularly when the number of available images is limited. In the work proposed by Balamurugan et al. [51], a method for the extraction of PRNU through three stages: filtering, estimation, and enhancement is proposed. Each stage utilizes various techniques to improve the accuracy of SCI. Experiments conducted on 300 natural images from six different camera models demonstrate the effectiveness of the approach, where the identification of the source camera is achieved by correlating the PRNU reference pattern with the noise residual model derived from the test image.

A summary of the PRNU-based SCI methods is presented in Table 3.

### Discussion

PRNU remains one of the most widely studied and utilized sensor-level fingerprints in the field of source camera identification (SCI). Its widespread appeal stems from several desirable properties: PRNU is unique to each sensor, stable over time, and embedded consistently across images taken by the same device. As shown in the reviewed studies, these characteristics make PRNU a powerful tool not only for linking images to cameras but also for organizing large image databases and detecting image tampering.

A recurring theme in PRNU-based research is the balance between identification accuracy and computational efficiency. Early works such as [31] demonstrate the feasibility of scalable PRNU matching by optimizing noise extraction and threshold-based clustering, enabling fast image grouping in large datasets. This reflects a practical orientation in PRNU-based SCI, which is particularly relevant in forensic triage scenarios involving massive amounts of visual data.

The use of PRNU in combination with statistical and machine learning classifiers, as in [1], reveals its potential as a discriminative feature for broader classification tasks, such as identifying camera brand and model. Although PRNU is inherently a device-level fingerprint, these results highlight its versatility and show that, when paired with appropriate feature extraction and modeling techniques, it can support more general camera profiling applications.

Importantly, PRNU has also been explored beyond traditional identification, including forensics tasks like image integrity verification and device linkage in manipulated or altered content [32]. This indicates a growing recognition of PRNU’s value in more complex forensic workflows. The ability to detect inconsistencies in PRNU across image regions makes it a candidate tool for forgery detection, source attribution in tampered media, and estimation of image acquisition conditions.

Despite these strengths, PRNU-based methods face several challenges. Extraction of clean PRNU signatures is sensitive to scene content, post-processing (e.g., compression, resizing, denoising), and the number of images available for reference fingerprint construction. Moreover, PRNU suffers from non-negligible interclass similarity for cameras of the same make and model, leading to increased false positives in certain scenarios. These limitations motivate further refinement of noise extraction pipelines and the development of robust similarity metrics.

While many works have demonstrated the effectiveness of PRNU-based identification within a specific dataset or under constrained conditions, the problem of cross-device generalization remains a major open challenge. In particular, the ability of PRNU to maintain high identification accuracy when applied to previously unseen devices of the same model or across different sensor types has not been systematically explored in the literature. Most existing studies evaluate performance within a closed-world setting, where the same camera instances are present in both training and testing phases. However, in real-world applications, a more realistic open-world scenario may involve unknown devices, firmware versions, or sensor variations, which can significantly affect PRNU stability and identification accuracy. Although some preliminary investigations into cross-sensor and cross-device generalization have been conducted [2], a unified evaluation framework and large-scale benchmarks for such settings are still missing. Addressing this gap is a crucial direction for future work in PRNU-based source attribution.

Additionally, the emergence of machine learning and hybrid frameworks opens new possibilities for enhancing PRNU utilization. For instance, deep networks trained to learn residual features from preprocessed images could improve the accuracy and robustness of PRNU-based SCI. Such integration remains largely unexplored and represents a promising direction for future research.

In summary, PRNU continues to serve as a cornerstone technique in the SCI landscape, providing strong performance and wide applicability. To maintain its relevance, ongoing research must address computational scalability, robustness to adversarial conditions, and integration with modern learning-based methods.

## 5. Deep Models

Deep learning offer advanced techniques for identifying digital cameras by analyzing unique patterns in images beyond traditional features. These methods utilize large datasets and neural networks to learn the cameras’ characteristics, making it possible to improve the accuracy and robustness of camera identification. This section covers the fundamentals of applying deep learning to source camera identification, including model training, feature extraction, and the advantages of these approaches in digital forensics.

In the work by Yao et al. [52], a robust multi-classifier based on CNNs for SCI is presented. It introduces an improved CNN architecture that automatically extracts relevant features from various camera models. The multi-classifier can simultaneously identify images from a wide range of camera models, achieving nearly 100.0% accuracy through majority voting. Its efficacy is further validated against common post-processing attacks, such as JPEG compression and noise, confirming its reliability.

The research presented by Tuama et al. in [30] introduces an SCI method utilizing CNN, which automates feature extraction and classification simultaneously. A preprocessing layer featuring a high-pass filter is applied to the input image before passing it into the CNN. The study evaluates the CNN with two types of residuals, with subsequent convolution and classification occurring within the network. The CNN outputs an identification score for each camera model. Experimental results demonstrate that this approach significantly outperforms traditional two-step machine learning methods. The performance of well-known object recognition CNN models, such as AlexNet and GoogleNet, is also analyzed for comparison.

In the work proposed by Rafi et al. [53], a DenseNet-based pipeline for SCI, addressing challenges posed by the absence of metadata and post-processed images is discussed. The method involves extracting 256×256-pixel patches from a labeled dataset and applying augmentations, including empirical mode decomposition (EMD). The extended dataset is used to train a neural network based on the DenseNet-201 architecture, combining output features from three different patch sizes 64×64, 128×128, and 256×256 pixels for final predictions. This robust approach achieved a state-of-the-art accuracy of over 99.0% on the Dresden Image Database across 19 models. Additionally, the pipeline demonstrated versatility by accurately detecting post-processing types with an accuracy of 96.7%, highlighting its applicability to various image forensic tasks.

The paper proposed by Marra et al. [54] presents a CNN-based method for iris sensor model identification. The proposed approach identifies the sensor model first and then maps features from one sensor to another. To minimize complexity and memory usage, a streamlined network architecture is utilized along with transfer learning to expedite training and address the challenges posed by limited training datasets. Experiments conducted on several public iris databases demonstrate that the proposed method outperforms existing state-of-the-art techniques for SCI. Additionally, the results indicate that improving the SCI process enhances iris sensor interoperability, which may lead to benefits for overall biometric recognition systems. The iris sensor identification is also discussed in [55]. The proposed approach could be especially useful for the task of double-checking user authentication, for instance, for iris biometric logging into the authentication system with the use of mobile devices.

In the work proposed by Freire-Obregón et al. [56], an SCI method for mobile devices utilizing deep learning techniques, specifically CNN is introduced. CNNs focus on automatically extracting features from data through layers of high-pass filters applied to input images, enabling them to learn noise patterns. The proposed CNN architecture aims to identify both the mobile device used to capture an image and the specific embedded camera within that device, achieving a high accuracy of 98.0%. The authors conduct a comprehensive analysis of different configurations of the architecture, using the MICHE-I dataset comprising images from various mobile device cameras. The experimental results demonstrate the robustness and efficacy of the proposed method in accurately detecting and identifying mobile camera sources.

The paper by Bondi et al. [7] proposes a data-driven approach using CNN. Unlike traditional methods, which, in general, require denoising images, the proposed CNN learns model-specific features directly from images. Testing on a dataset with 18 camera models demonstrates that this method outperforms state-of-the-art algorithms for classifying 64×64 pixels color patches.

A deep learning approach based on residual neural networks (ResNet) is presented in the work by Chen et al. [57]. This approach utilizes both low-level and high-level features to improve the identification accuracy. The proposed framework was tested on tasks including brand, model, and device attribution. The results demonstrate that the method enhances identification performance across all tasks, establishing it as a highly effective tool.

The work proposed by Yang et al. [58] addresses the challenges of the SCI for small-size images. The authors propose a content-adaptive solution using fusion residual networks (FRN) to improve identification accuracy. The FRN consists of three parallel residual networks, each differing in the convolutional kernel size used in the preprocessing layer. The features learned from the last residual blocks of these networks are then combined and fed into a softmax classifier for final identification. The design of the residual networks focuses on enhancing feature representation from the input data, utilizing convolutional operations in the preprocessing stage along three residual blocks. The images are categorized into three subsets based on content characteristics: saturation, smoothness, and others. For each subset, they are trained by separate FRN, utilizing transfer learning. The experimental results indicate that the proposed method achieves satisfactory performance across different levels of source camera identification, including brand, model, and device levels, demonstrating its efficacy in different scenarios.

A patch-level SCI method using a convolutional neural network (CNN) is introduced in the paper by Liu et al. [59]. The approach selects representative patches based on multiple criteria to improve training data diversity. A multiscale deep residual prediction module is used to soften the influence of scene content. Additionally, a modified VGG network is utilized for identification at brand, model, and instance levels. Experimental results demonstrate that the proposed method outperforms existing state-of-the-art algorithms, highlighting its efficacy in real-world applications. CNNs are also discussed in [60], where the proposed approach features a network architecture comprising an input layer, three convolutional layers with max pooling and normalization, two fully connected layers, and a softmax classifier. It processes small-sized patches of original images to reduce the dependency on large training datasets from specific cameras. A local-to-global strategy is employed, utilizing majority voting among the image patches to enhance identification accuracy. Experimental results demonstrate that this method achieves an accuracy of up to 99.8%, validating the effectiveness of the majority voting mechanism. Additionally, a support vector machine (SVM) classifier trained on deep convolutional features extracted from the network outperforms the softmax classifier.

The paper proposed by Sychandran et al. [61] introduces an architecture for identifying the SCI, utilizing a combination of convolutional layers and residual blocks. The architecture includes batch normalization, a fully connected layer, and a softmax layer, learning and extracting features for distinguishing between model and sensor-level patterns. By taking multiple patches from each image, the sample size is increased, leading to high accuracy results on the MICHE-I dataset, achieving 99.5% accuracy for model-level identification and 96.0% for sensor-level identification, exceeding state-of-the-art methods. The architecture also shows strong performance on the Dresden and VISION datasets. Additionally, a technique is proposed to identify images from unknown camera models by establishing a threshold for output prediction scores.

In the work by Bernacki [62], the impact of the activation function on the speed of CNN learning is discussed. The authors explore several existing approaches using CNNs and a key finding is the replacement of the ReLU activation function [63] with the SELU activation function [64], which significantly accelerates learning. The paper includes a comparison of identification accuracy across various methods, based on experiments conducted on a comprehensive dataset of images from contemporary cameras.

The work presented by Marra et al. in [65] considers a method for SCI by analyzing traces left by in-camera processes unique to each model. The authors focus on blind features derived from image residuals, specifically extracting local features using co-occurrence matrices of selected neighboring pixels. These features are then utilized to train a support vector machine (SVM) classifier. Experiments conducted on the Dresden Image Database demonstrate that this approach achieves state-of-the-art performance in accurately identifying camera models.

The research presented by Tsai et al. in [66] employs a support vector machine (SVM) approach, utilizing various image-related and hardware-related features, along with decision fusion techniques. Additionally, the study investigates the optimal feature subset to maximize accuracy, aiming to enhance the efficacy of the SCI.

In the work by Zheng et al. [67], a module combining residual and squeeze-and-excitation (SE) elements is proposed. To optimize resource use and improve performance, the authors introduce the adaptive dual-branch fusion network (ADF-Net), designed to identify digital image sources efficiently. The ADF-Net utilizes a bottleneck residual module to facilitate the transfer of shallow features, ensuring that images retain important source characteristics without excessive compression. Additionally, a channel attention mechanism enhances the significance of effective feature channels, boosting overall network performance. The results demonstrate a high accuracy, achieving 99.3% on the Dresden Image Database.

An ensemble classifier that combines software-related, hardware-related, and statistical features from images is proposed in the work by Wang et al. [68]. The experimental results indicate that this approach achieves nearly 100.0% accuracy in identifying camera brands and models, while also outperforming baseline methods in distinguishing individual cameras. This advancement enhances the reliability of forensic investigations in associating images with their source cameras.

The work proposed by Wang et al. [69] discusses an approach for SCI in scenarios with limited training samples. The authors propose an approach called multi-distance measures and coordinate pseudo-label selection (MDM-CPS), utilizing semi-supervised learning to iteratively expand and refine the labeled database. By minimizing the impact of noisy pseudo-labels during training, the method enhances the reliability of predictions for pseudo-labeled samples. Comprehensive experiments demonstrate that MDM-CPS achieves a reliable performance in few-shot scenarios on datasets including Dresden Image Database and VISION.

In the paper by Zhang et al. [70], an approach called tri-transfer learning (TTL) is presented. This algorithm addresses performance issues when training and testing sets come from different statistical distributions due to double compression. TTL combines transfer learning and tri-training, using a transfer learning module to enhance identification performance with fewer training samples and a tri-training module to assign pseudo-labels to unlabeled instances. Experiments on the Dresden Image Database demonstrate that TTL significantly outperforms existing methods in mismatched camera model identification, offering higher identification accuracy.

In the paper presented by Lu et al. [71], an algorithm that employs multi-scale feature fusion is proposed. This method extracts local features from different scales of feature maps and fuses them to create a comprehensive representation. The fused features are then processed by a classification network, enhanced by transformer blocks and graph convolutional network (GCN) modules. The representative experiments confirm the efficacy of the proposed approach.

The paper by Wang et al. [72] addresses the challenge of SCI based on few-shot scenarios. It is proposed a method called Prototype Construction with Ensemble Projection (PCEP). This approach extracts diverse features from limited datasets and employs semi-supervised learning to build prototype sets. SVM classifiers are then trained using these prototypes, with the probabilities of image samples serving as final projection vectors. The classification results are derived through ensemble learning voting. Comprehensive experiments on benchmark databases like Dresden Image Database, VISION, and SOCRatES demonstrate that the PCEP method effectively utilizes image information from few-shot datasets, achieving satisfactory performance.

A framework utilizing both frequency and spatial features is proposed in the work by Jaiswal et al. [73]. Using discrete wavelet transform (DWT) and local binary pattern (LBP) techniques, the method extracts diverse feature vectors from augmented images, which are then classified with multi-class models like SVM, LDA, and *k*-NN. Comparative experiments indicate that this hybrid feature approach significantly improves the identification accuracy.

The study described by Lai et al. in [74] addresses the case of image acquisition and editing issues. It employs image interpolation to determine characteristic values of images and utilizes an SVM for classification, aiming to enhance the efficacy of SCI. Various camera brands and models were tested in the experimental setup. The results demonstrate that the proposed method achieves a recognition rate of up to 90.0%, particularly with the additional use of a wave filter, indicating its reliability.

In the work by Wang et al. [75], a method called Siameformer is introduced. While traditional SCI methods typically assume that images are unaltered, images shared on social networks are often transformed, such as compression application or resizing, which degrade the identification accuracy. To address this issue, the proposed approach combines the CNNs and vision transformers (ViT) within a Siamese network architecture, enhanced by multi-head attention mechanisms, to improve robustness in identifying image sources. The experiments on Dresden Image Database, VISION, and Forchheim demonstrate the model’s high performance in real-world scenarios, suggesting Siameformer as an effective solution.

The work proposed by Alhussainy et al. [76] proposes two approaches to improve the SCI. The first method uses machine learning with features like co-occurrence matrices, CFA interpolation patterns, and JPEG domain statistics, achieving high accuracy. The second approach employs CNNs with a preprocessing high-pass filter layer, allowing for automatic feature extraction and classification with strong results. Comparisons with traditional methods show the CNN model’s efficacy in enhancing identification accuracy.

A machine learning approach that extracts the following sets of features: co-occurrence matrices, features related to color filter array (CFA) interpolation arrangements, and conditional probability statistics based on images is described in the work by Tuama et al. [77]. These high-order statistics enhance the identification rate. The method was tested using 14 camera models from the Dresden Image Database, employing a multi-class support vector machine (SVM) classifier. The results indicate that this approach overcomes traditional methods utilizing the PRNU, achieving higher accuracy in SCI.

The paper by Bharathiraja et al. [78] describes the enhancement of digital image forensics by automating the process of SCI. The authors propose a deep learning framework that learns the intrinsic signature of a specific camera model. The framework consists of two main components: a residual noise feature extractor (RNFE) that analyzes degraded images using a U-Net to extract noise patterns, and a feature modulator (FM) that processes these patterns into an embedding vector via a CNN. The model is trained using a triplet loss function to ensure that images from the same camera are closer in feature space than those from different cameras. The experimental results demonstrate the CNN’s efficacy, achieving a 97.6% F-score and 97.0% recall, compared to state-of-the-art methods. This architecture is positioned as a framework for learning the SPN fingerprint of camera models.

In recent works applying Transformer architectures to source camera identification, multiple feature extraction strategies are often employed to improve performance. For instance, some approaches utilize hybrid pipelines, where both spatial domain features (such as image patches or noise residuals) and frequency domain representations (e.g., DCT or wavelet coefficients) are combined as model inputs. Other strategies involve fusing handcrafted statistical descriptors with learned deep features before feeding them into the Transformer layers. These multi-feature pipelines aim to exploit the complementary nature of various signal components, such as global structural patterns and local sensor noise which may not be effectively captured by a single feature representation. However, due to the novelty of applying Transformer models in this domain, systematic comparisons between single- and multi-feature approaches remain scarce, and more research is needed to fully understand their respective advantages [79].

A summary of the SCI methods utilizing deep models is presented in Table 4.

### Discussion

The studies discussed in this section illustrate the growing potential of deep learning for source camera identification. A common feature among these approaches is the ability to automatically extract relevant features from raw image data, often outperforming traditional handcrafted techniques. In particular, the use of convolutional neural networks (CNNs) has enabled end-to-end learning pipelines that simultaneously handle feature extraction and classification, simplifying the processing chain.

One recurring strategy is the inclusion of preprocessing layers, such as high-pass filters or residual extraction, which aim to enhance device-specific patterns before feeding the image into the network. This shows a trend toward embedding domain knowledge into the network architecture, rather than relying solely on raw data-driven learning. Furthermore, some methods utilize ensemble strategies or majority voting mechanisms, which have proven effective in improving robustness and classification accuracy, especially in multi-camera setups.

Despite these advances, certain limitations remain visible across the reviewed works. For instance, most deep learning-based methods are evaluated under relatively controlled conditions, using datasets with limited diversity in terms of camera models, scenes, and post-processing. This raises concerns about generalization to real-world conditions, where compression, scaling, or adversarial perturbations are common. Moreover, while deep networks achieve high-performance metrics, they often do so at the cost of interpretability, which is critical in forensic applications where decisions must be explainable.

In summary, the deep learning approach offers a powerful and scalable solution for SCI. However, to transition from promising research to practical forensic tools, future studies should prioritize cross-dataset validation, adversarial robustness, and interpretability of decisions. Additionally, integrating classic statistical techniques or SPN-based priors with modern deep architectures could lead to more reliable and transparent identification systems.

## 6. Identification by Analyzing Aberrations

Aberration analysis includes techniques used to identify digital cameras based on characteristic optical distortions unique to each device’s lens and sensor configuration. These aberrations, such as chromatic and geometric distortions, lens vignetting, and chromatic aberration (CA), result from imperfections in lens alignment and sensor design, producing consistent patterns in images. This section discusses the issue of aberration-based digital camera identification and its effectiveness in linking images to their source devices.

The work proposed by Choi et al. [80] considers the SCI by utilizing the intrinsic lens radial distortion unique to each camera, which serves as a fingerprint for the images produced. Given that most digital cameras use spherical lenses to reduce manufacturing costs, the resulting radial distortions can be effectively analyzed. The authors extract aberration parameters from each image and employ them to train and test a support vector machine classifier. Extensive experiments involving five different cameras demonstrate a high accuracy rate for such identification methods. Additionally, the study examines how error rates vary with images captured at different optical zoom levels, highlighting the robustness of the approach.

In the work by San et al. [81], the focus on intrinsic lens aberration, and a lens radial distortion as a distinguishing feature for camera classification is presented. Instead of relying solely on image intensity patterns, this method extracts parameters related to pixel intensities and aberration measurements to train a classifier, which then identifies the camera source with higher accuracy. Experimental evaluation indicates the efficacy of this approach, raising accuracy from 87.0% to 91.0% compared to methods based only on intensity.

The research described by Yerushalmy et al. in [82] proposes a method for detecting forgery in digital photographs without the need for additional data, such as digital watermarks, or reference images for comparison or training. The method focuses on exploiting optical and sensing system effects, particularly using image artifacts caused by chromatic aberrations as indicators to assess the authenticity of images. The core idea is that certain image features serve as proof of authenticity. These features are sensitive to manipulations and are challenging to replicate synthetically, typically remaining invisible and not affecting the overall image quality.

In the work proposed by Yu et al. [83], the challenges in lens identification using chromatic aberration patterns, specifically the difficulty in distinguishing between different copies of the same lens are discussed. It overcomes two major hurdles: first, by replacing the conventional checkerboard target, prone to misalignment, with a white noise pattern for capturing images. Second, it emphasizes the importance of utilizing the lens focal distance, which has been overlooked in previous studies. These improvements lead to the generation of stable chromatic aberration patterns that can effectively differentiate between copies of the same lens. This approach enables reliable lens identification within a large database of lenses.

The estimation of parameters related to lateral chromatic aberration (LCA) is described in the work by Yan et al. [84]. The LCA is a phenomenon where different wavelengths of light fail to focus at the same position on the sensor. It differentiates between longitudinal aberration, which affects focus distance, and lateral aberration, which alters the focus position. The authors propose a method that maximizes the mutual information between the corrected red and blue channels and the green channel to estimate the parameters of LCA. These parameters are then utilized as input features for a support vector machine classifier to identify the source cell phone of images. By focusing on a specific portion of the image for parameter estimation, the algorithm significantly reduces runtime complexity while maintaining high accuracy.

In the paper proposed by Bernacki [85], the SCI is realized through the analysis of sensor artifacts present in images. While many algorithms using wavelet-based denoising to calculate the SPN are robust and efficient, they may be time-consuming. The authors propose an approach that focuses on optical system defects, specifically vignetting and lens distortion, to identify camera brands and distinguish between different models. Experimental evaluations were conducted on 60 devices, on a total number of 12,051 images, with the support of the Dresden Image Database. The proposed methods eliminate the need for denoising, significantly improving processing speed compared to state-of-the-art techniques.

The study by Dirik et al. [86] addresses the issue of sensor dust in digital single-lens reflex (DSLR) cameras. It occurs when dust particles settle on the sensor, creating distinctive patterns that appear as artifacts in captured images, particularly noticeable at smaller aperture values. Since these dust patterns remain unchanged until the sensor is cleaned, they can be utilized for SCI. The proposed method detects dust specks by analyzing intensity variations and shape features to construct the dust pattern associated with a specific DSLR camera. The experimental results demonstrate that this approach effectively identifies the source camera with very low false positive rates, offering a reliable solution for camera identification in forensic applications. An extension of this work is presented in [87], where detecting and matching the characteristics of dust spots, utilizing a Gaussian intensity loss model, and analyzing shape properties are discussed. To reduce false detections, the method also considers lens parameter-dependent features of the dust spots. The experimental results demonstrate that this detection scheme effectively identifies the source DSLR camera with low false positive rates, even in images that have undergone heavy compression and downsampling.

A summary of the SCI methods utilizing aberrations analysis is presented in Table 5.

### Discussion

Aberration-based methods for source camera identification represent a distinct and complementary approach to noise-based techniques such as PRNU or SPN. Instead of analyzing sensor-level noise patterns, these methods rely on optical distortions that arise from imperfections in the lens and sensor alignment, which are inherent to each camera’s physical construction. As such, aberrations serve as device-specific yet scene-independent markers, providing an alternative and often more interpretable path to forensic attribution.

The studies discussed highlight the diversity of optical aberrations that can be utilized, with particular emphasis on radial distortion and chromatic aberration. In [80], radial distortion is shown to be both measurable and sufficiently unique to act as a camera fingerprint. This approach demonstrates strong classification accuracy across multiple cameras, and crucially, maintains robustness across different zoom levels—an important factor given the variability introduced by optical settings in practical image capture scenarios.

Further enhancement of classification accuracy is demonstrated in [81], where hybrid features combining pixel intensities with aberration parameters improve performance. This finding suggests that aberration features do not necessarily need to function in isolation; rather, they may synergize with other image-based descriptors to support more accurate and resilient camera classification frameworks.

A particularly noteworthy contribution comes from [82], which extends aberration analysis beyond identification and into image integrity verification. Here, chromatic aberrations are treated as subtle, high-fidelity fingerprints that are hard to forge or simulate. Their presence or absence can serve as a form of passive authentication, with the potential to detect content manipulation. This shifts the role of aberration features from attribution to tamper detection, opening a promising dual-use potential within digital forensics.

Despite these advantages, aberration-based methods are not without limitations. One of the key challenges is variability introduced by image content, camera settings (e.g., zoom, aperture), and scene geometry, which may influence the expression or detectability of aberrations. Moreover, unlike sensor noise, optical aberrations may be altered or partially corrected by modern in-camera processing pipelines, especially in smartphones and higher-end digital cameras. This could reduce the consistency of aberration features across devices and over time.

Furthermore, while aberration-based techniques are typically more explainable and grounded in physical optics, they often require careful geometric calibration or modeling, and the feature extraction process can be sensitive to camera pose, subject distance, or framing, especially for distortion-based methods.

Nevertheless, these techniques offer a valuable non-intrusive, and reference-free approach, which can function without the need for large image sets or prior training. When combined with sensor-level analysis, aberration-based identification can strengthen the evidentiary value of forensic conclusions, providing orthogonal evidence streams for image attribution or authentication.

In conclusion, optical aberration analysis offers a promising and underexplored avenue in the SCI domain. Its utility is evident not only in source identification but also in image integrity verification, especially in scenarios where traditional noise-based fingerprints may be weak or absent. Future research should focus on developing robust, content-independent descriptors of aberrations, and on integrating optical and sensor-level cues into unified forensic frameworks.

## 7. Statistical Methods

The source camera identification may be realized also with the use of statistical methods. By correlation analysis, these methods help to detect consistent features within images that can link them to their source cameras. In this section, we explore some statistical techniques for SCI.

A statistical test for SCI from JPEG images, focusing on the discrete cosine transform (DCT) domain, is presented in [88]. By analyzing how DCT coefficients’ statistics change due to varying sensor noises and in-camera processing algorithms, the authors utilize a state-of-the-art DCT coefficient model to define camera fingerprints through two key parameters. First, they introduce a likelihood ratio test (LRT) for ideal scenarios, and second, generalized likelihood ratio tests (GLRTs) for practical situations with unknown parameters. The experiments on simulated and real JPEG images confirmed the efficacy of this approach for an accurate SCI.

The study proposed by San et al. [89] addressed the examination of the differences in JPEG compression processes among various digital cameras. While JPEG is a standard compression algorithm, its size and quality trade-offs depend on both manufacturers and user preferences. The authors focus on identifying specific features that differentiate these trade-offs across different camera models. The evaluation of the efficacy of different JPEG compression levels in classifying images from multiple cameras is performed by simulations. The results indicate that the identified features are valuable for enhancing the success rate of the SCI.

The paper by Thai et al. [90] presents a statistical test using a generalized noise model. Starting from a heteroscedastic noise model, the approach uses the nonlinear effects of gamma correction to better characterize natural images in TIFF or JPEG formats. Building on previous work focused on RAW images, the authors utilize parameters from the generalized noise model as a camera fingerprint. The identification process is framed within hypothesis testing theory, employing the likelihood ratio test (LRT) in an ideal scenario with known parameters. To address practical challenges with unknown parameters, two generalized likelihood ratio tests are introduced to maintain a specified false alarm probability while achieving high detection performance. The results from real JPEG images demonstrate the efficacy of the proposed method.

In the work by Mieremet et al. [91], the exploration of the PRNU, focusing on the correlation values of mismatches between images captured by different cameras is considered. While mismatch correlation values between the estimated PRNU and a noise residual are typically close to zero, they never reach exactly zero. The authors present a formula to a priori estimate the typical range for such mismatch correlation values, which can serve as a decision rule for SCI.

Agarwal et al. [92] proposed a fingerprint sensor identification algorithm based on features like Haralick, entropy, statistical, and image quality attributes. Evaluated on a dataset of 30,000 images across 15 sensor classes, the algorithm achieves 96.0% accuracy and processes each image in under 10 milliseconds. In the study [93], an algorithm that combines bacteria foraging (BF) feature selection, guided by an SVM fitness function, with a fusion of features, including block image statistical measure (BISM), high-order wavelet entropy (HOWE), texture measure (TM), single-level multi-orientation wavelet texture (SlMoWT), and image quality measure (IQM) is presented. These selected features are then classified to identify the iris sensor. Additionally, the study contributes two new multisensor iris databases with 6000 images from over 150 subjects. The proposed algorithm is highly efficient, achieving over 99.0% accuracy across multiple databases.

A method based on texture features from selected color models and channels to identify source cameras even distinguishing between individual devices of the same brand and model is introduced in the paper by Xu et al. [94]. The method is robust to content-preserving manipulations and geometric distortions, such as JPEG compression, noise addition, rotation, and scaling. The experimental results show that it exceeds state-of-the-art methods in both accuracy and robustness.

A summary of the statistical SCI methods is presented in Table 6.

### Discussion

Statistical methods for source camera identification provide a systematic and often robust means of analyzing images based on probabilistic and mathematical models. These approaches exploit subtle statistical regularities introduced by in-camera processing, such as compression, quantization, or transformations in the frequency domain.

One of the primary strengths of statistical methods is their resilience to image content. Since they focus on underlying distributions and not on scene details, they can be applied to a wide variety of images without requiring consistent visual features. Additionally, many statistical approaches are computationally efficient, making them suitable for large-scale image classification tasks or forensic triage.

Fusion strategies between handcrafted (statistical) and learned features have recently gained attention in camera identification. In such approaches, shallow descriptors, such as local binary patterns, co-occurrence matrices, or noise residual statistics, are combined with deep neural network outputs at either the feature or decision level. This hybrid strategy benefits from both domain-specific prior knowledge and the powerful abstraction capabilities of deep learning. While some studies focus on concatenating feature vectors before classification, others propose multi-branch architectures or ensemble systems. For instance, fused handcrafted PRNU features with CNN-based embeddings to improve robustness against post-processing operations. Such strategies can improve generalization and interpretability, especially when training data are limited or heterogeneous.

A common application area is the analysis of JPEG compression artifacts. Cameras, even when using the same compression standard, may apply it in slightly different ways, leaving identifiable patterns. These differences can often be detected through analysis of quantization noise, coefficient distributions, or residuals, providing a form of fingerprinting even when other cues are lost.

However, these methods have some limitations. They typically rely on the presence of original compression or encoding traces, which may be degraded or eliminated through recompression or aggressive post-processing. Furthermore, they may offer less device-specific resolution, being more effective at distinguishing between camera models or brands rather than individual units.

Another challenge lies in the need for well-calibrated models. Many statistical techniques assume a certain regularity or homogeneity in the data, and their performance can drop if the test conditions deviate significantly from those used during training or calibration. Moreover, changes in firmware or image processing pipelines over time can affect the stability of statistical features.

Despite these challenges, statistical methods remain an important element in the broader landscape of camera identification. They are particularly valuable when traditional sensor-based fingerprints are unavailable or unreliable, and can complement other methods by providing orthogonal information. When combined with noise-based or geometric features, statistical approaches can enhance overall identification accuracy and robustness.

In summary, statistical techniques offer a flexible and practical framework for digital camera identification, especially in scenarios where content-independent, efficient, and scalable analysis is required. Their integration into multi-feature forensic systems can significantly strengthen evidentiary conclusions, particularly when dealing with compressed or degraded images.

## 8. Robustness and Adversarial Attacks

Assessing the robustness of identification methods is essential to determine their reliability in forensic applications. Robustness testing evaluates how well camera identification methods perform under various conditions, such as compression, noise, and post-processing, which may alter identifying features in images or videos. Adversarial attacks on the SCI methods refer to the ability to manipulate or alter images or videos in such a way that they deceive algorithms designed to recognize and attribute them to specific devices. This can involve techniques like adding noise, altering image features, or injecting malicious content into the image. In this section, we discuss both the robustness of SCI methods, as well adversarial attacks that may impersonate cameras.

In the study proposed by Akshatha et al. [95], a feature-based approach to improving the PRNU robustness against random noise and simple image manipulations is proposed. The method extracts PRNU using a wavelet-based denoising technique and represents it with higher-order wavelet statistics (HOWS), which are invariant to image manipulations and geometric changes. These features are then classified using support vector machines and validated through a ten-fold cross-validation technique. Experiments on images captured by various mobile phone cameras demonstrate that the proposed method effectively identifies the source camera, even among similar models, while maintaining robustness against common manipulations.

The paper proposed by Bernacki [96] considers the issue of altered images, particularly in the context of social media where numerous photographs are shared. The study focuses on evaluating the robustness of CNNs in identifying cameras, even when images are of poor quality due to factors like Poisson noise, Gaussian blur, random noise, or the removal of the least significant bit. The experimental results from two large datasets, including the Dresden Image Database, demonstrate that the proposed method maintains high accuracy in recognizing noisy images, comparable to that of normal images.

A framework for precise and robust camera model identification by utilizing demosaicing information from camera output images is proposed in the work by Chen et al. [97]. The framework extracts a variety of intra-channel and inter-channel color value correlations resulting from the demosaicing process. This is achieved by applying multiple baseline demosaicing algorithms to the query image, collecting both linear and nonlinear demosaicing residuals. Co-occurrence matrices are then computed using specially designed geometric patterns to capture color value dependencies based on the color filter array (CFA) structure. The extracted correlations are utilized in a multi-class ensemble classifier for camera model identification. The experimental results demonstrate that the framework achieves a high accuracy of 98.1% across 68 camera models and exhibits strong robustness against post-JPEG compression and contrast enhancement.

In the work proposed by Samaras et al. [98], the impact of various image processing operations is investigated. It highlights that artifacts such as pixel defects, CCD response irregularities, and black current noise leave distinctive marks in images, enabling effective camera identification. The sensor fingerprint is derived from images captured by a specific device, and a similarity measure is used to link an image to its originating camera. However, image processing techniques like gamma correction, contrast enhancement, histogram equalization, and white balance can alter the properties of the detection statistics, potentially compromising fingerprint detection. Through experimental analysis, this study aims to quantify the robustness of fingerprint detection methods against these image processing operations.

The work depicted by Rosenfeld et al. [99] explores the robustness of PRNU-based camera identification. The test images are subjected to common processing operations, such as denoising, recompression, and out-of-camera demosaicing. The experimental evaluation focuses on the identification process, assessing whether these standard practices lower the ability to accurately identify the source camera from processed images.

The research presented in a paper by Goljan et al. [100] considers the scenario where images have been cropped and scaled. The detection of the PRNU is framed within a hypothesis testing framework, treating it as a two-channel problem, and employing a generalized likelihood ratio test to derive a detector. A brute force approach is introduced to identify the scaling factor, followed by a refined search for accuracy. Cropping parameters are determined by maximizing the normalized cross-correlation between two signals. The efficacy and limitations of this technique are evaluated on images subjected to various cropping and scaling transformations, including digital zoom. Furthermore, the study illustrates how sensor noise can aid in reverse-engineering in-camera geometrical processing and recovering from subsequent geometric transformations, which could enhance digital watermark detection synchronization.

The paper described by Lin et al. [101] examines the vulnerability of deep neural networks (DNN) to adversarial attacks. Such attacks can significantly manipulate identification outcomes through imperceptible noise added to the images. The authors analyze the feature extraction mapping in DNNs and identify that oscillations in this mapping contribute to the model’s susceptibility. To counteract this issue, a design principle for robust SCI, emphasizing locally smooth mappings that uphold information monotonicity is proposed. This is achieved through a defensive scheme that minimizes the Kullback–Leibler divergence (KLD) between local statistic coordinates across two manifolds. To enhance the method’s usability, a pre-defense network (PDN) is implemented and trained via a two-phase strategy, which optimizes for robustness, accuracy, and portability. Experimental results on the Dresden Image Database demonstrate that this defense mechanism significantly improves the robustness of DNN-based SCI models against adversarial attacks. Additionally, the PDN shows effective defense capabilities when applied to other DNN-based SCI models without requiring further retraining.

In the works by Martin et al. [102,103], the extraction of camera fingerprints understood as PRNU is addressed. The study evaluates a set of attacks against a PRNU-based SCI system, assessing their effectiveness on both still images and videos. Each attack is designed to minimally alter image quality while attempting to deceive PRNU detectors. The success of these attacks is measured by their impact on the SCI system’s error rate. Key findings include a comprehensive testing framework involving over 2000 test images and the identification of several effective attacks that can compromise PRNU-based identification methods.

Adversarial attacks against the camera’s fingerprint classifiers have become a growing concern in computer vision, particularly when it comes to fooling image classifiers by adding noise to the images [104,105,106]. While much of the research focuses on manipulating the photos, the challenge of attacking object detection models and conducting adversarial attacks in the physical world remains underexplored. Moreover, with the rapid advancement of synthetic media generation, creating realistic fake images has become increasingly accessible, posing significant risks for spreading misinformation. As digital forgeries become more common, multimedia forensics must develop reliable detection methods, particularly for SCI tasks. However, these techniques are vulnerable to adversarial perturbations, which can mislead forensic analysis even when changes are imperceptible to the human eye.

In the paper proposed by Cozzolino et al. [107], the vulnerabilities of the SCI detectors are discussed. A generative adversarial network-based (GAN) method that injects camera-specific traces into synthetic images, tricking detectors into classifying them as genuine is proposed. The approach effectively deceives both the camera model and GAN-image detectors, with only sample images from the target camera required and no prior knowledge of detector models. The experiments validate the approach across diverse conditions, highlighting its reliability.

The approach presented in the paper presented by Chen et al. [108] introduces an anti-forensic framework based on GANs that can falsify an image’s camera model traces without noticeable artifacts. The method adapts depending on the attacker’s knowledge level: in a white-box scenario, where the attacker has full access to the forensic model, the model is used directly in training. In a black-box scenario, a substitute network that mimics the classifier’s decisions for adversarial training is created. Tests on multiple CNN-based classifiers confirm the method’s efficacy in both scenarios while preserving high image quality and generalizing across different camera models.

In the work by Sameer et al. [109], a CNN-based approach designed to detect if an image has undergone any counter-forensic anonymization is discussed. The model addresses three primary anonymization attacks, seam carving, fingerprint copying, and adaptive PRNU denoising, identifying both tampering presence and type through multiclass classification. The experiments demonstrate that the proposed model achieves high detection accuracy and avoids overfitting which allows for practical applications.

In the work presented by Yang et al. [110], an approach to launching physical adversarial attacks specifically targeting object detection models is proposed. Real metal objects designed to deceive detection models are created. The experiments, conducted in both indoor and outdoor environments, demonstrate that these physical adversarial objects successfully fool widely-used object detection models, including SSD, YOLO, and Faster R-CNN.

The article described by Li et al. [111] proposes the manipulation of the camera itself to fool deep classifiers across a wide range of objects. It is shown that by placing a specially designed, primarily translucent sticker over the camera lens, it is possible to induce universal perturbations in the captured images. These perturbations are subtle and difficult to detect, yet they cause the camera to misclassify target objects as a different, intended class. Experiments depict that such physically realizable attacks can deceive ImageNet classifiers with a targeted success rate of 49.6%.

Traditional adversarial attacks typically generate one-to-one noise, failing to learn the fingerprint information of the images. To address this issue, in the paper by Wang et al. [112] two advanced attack methods are proposed: the fingerprint copy-move attack and the joint feature-based auto-learning attack. To evaluate the efficacy of these attacks, a robust defense mechanism called relation mismatch, which enhances the differentiation capabilities of classifiers within the same network is considered. Extensive experiments demonstrate that relation mismatch outperforms other methods in detecting adversarial examples, and the proposed fingerprint-based attacks exhibit remarkable attack transferrability to unknown samples.

In [113,114], the investigation of the scenario where an adversary attempts to estimate the sensor fingerprint from a set of images and then superimposes it onto an image from a different camera to falsely attribute it to an innocent party is considered. The study proposes a reliable method for detecting such fake fingerprints under mild and general assumptions about the adversary’s resources and the victim’s countermeasures. Experimental results show that successfully embedding a fake sensor fingerprint into an image without leaving detectable traces is far more difficult than previously believed, highlighting the robustness of forensic detection methods against this type of attack. To detect such attacks, a method called the triangle test is introduced in [115]. Moreover, the exploration of whether more sophisticated attack strategies could bypass the triangle test countermeasure is examined. The experimental results demonstrate how attackers can improve their methods to successfully challenge the triangle test.

Li et al. [116] proposed an enhanced fingerprint-copy attack by superimposing the estimated fingerprint onto the target image in a dispersed, block-wise manner, using random and partially stolen images. Moreover, a method to adjust the strength of the superimposed fingerprint based on objective image quality is also introduced. This reduces the non-PRNU component’s effect on the triangle test, making such an improved attack harder to detect. Experiments on 2900 images from four cameras demonstrate that the proposed method effectively fools camera identification and significantly impairs the triangle test’s performance. Another attack scenario on the PRNU is described in [117].

In the research by Quiring et al. [118], an SCI based in adversarial environments with asymmetries, particularly focusing on fingerprint-copy attacks is examined. In this scenario, the attacker has access to JPEG images, while the defender can utilize uncompressed images. This setup introduces the concept of fragile sensor fingerprints, which are only accessible to the defender and are lost during lossy compression. Experiments with seven different cameras show that the attack can be reliably detected as long as high-quality images are not made publicly available.

An improved statistic for the pooled version of the triangle test is introduced by Barni et al. [119]. It is used to counter the fingerprint-copy counter forensic attack targeting PRNU-based camera identification. The proposed statistic differs from the original test by utilizing the one-tailed nature of the correlation test. It assigns different weights to positive and negative deviations from the expected correlation value between the image under analysis and the candidate images (those suspected of being used in the attack). The experimental results demonstrate that this statistic overcomes the original triangle test, particularly where the fingerprint-copy attack involves a large number of images, and the image under test is of a small size.

Marra et al. [120] considered the exploration of the robustness of CNNs for the SCI against adversarial attacks. The study evaluates several CNN architectures and attack strategies, considering scenarios where the attacker has either complete knowledge of the network or only access to the training set. It also examines the impact of both original and JPEG-compressed images, simulating conditions typical of social media environments. Experiments conducted on a representative dataset containing images from 29 different camera models confirm the efficacy of the proposed method and also show the limitations of CNN-based methods in this context.

Hsu et al. [121] proposed a fully automated method to detect prepared digital images by examining consistency in physical characteristics across different image regions. Specifically, it uses the camera response function (CRF), which maps input light to output intensity, as a fundamental feature. The method begins by segmenting an image into arbitrarily shaped regions and estimating a CRF for each segment based on local irradiance points. To identify splicing, CRF cross-fitting, and local image features are applied to boundary segments between regions, with scores feeding into statistical classifiers to assess image authenticity. Experiments show 70.0% precision and recall across datasets, highlighting boundary anomalies as a key factor in splicing detection.

A summary of the methods for testing robustness and adversarial attacks on SCI is presented in Table 7.

### Discussion

The robustness of camera identification methods is crucial for their practical application in forensic contexts, where the integrity of digital images cannot always be guaranteed. A method’s robustness refers to its resilience when faced with common challenges such as image compression, noise, or post-processing alterations. Given that real-world images are often subject to these factors, testing and understanding how identification techniques perform under such conditions is necessary to assess their effectiveness in varied environments.

One of the key challenges in forensic identification is the impact of image manipulations, which can degrade or obscure identifying features. Techniques such as compression, noise addition, and geometric transformations (e.g., cropping or resizing) are often used for tampering with images. These modifications may alter the image content in ways that interfere with the accuracy of traditional forensic methods. Robustness testing involves simulating these alterations to determine how well a method can maintain accurate identification despite these disruptions.

A notable approach to improving robustness, particularly for sensor-based methods like PRNU, is the use of denoising techniques or feature extraction methods that are less sensitive to common manipulations. For example, utilizing higher-order wavelet statistics (HOWS) in conjunction with PRNU extraction helps lower the effects of noise and geometric transformations, ensuring that the core identifying features remain intact. Such methods allow for accurate identification even when images are degraded or manipulated, such as through random noise or simple geometric changes.

However, the potential for adversarial attacks represents a significant concern in the security of digital forensic methods. Adversarial attacks are designed to deceive the camera identification systems, often by injecting carefully crafted noise or modifying image characteristics in subtle ways that cause the system to misidentify the source device. This poses a challenge for any SCI method, as attackers can exploit weaknesses in the algorithms to impersonate specific cameras or prevent accurate identification.

The issue of adversarial manipulation highlights the vulnerability of even sophisticated forensic techniques to deliberate tampering. The use of generative techniques to alter image features, inject noise, or even create entirely synthetic images has become a concern, especially with the rise of deepfake technologies. These methods, if successful, can undermine the reliability of traditional forensic approaches and call into question their effectiveness in real-world scenarios.

Despite these challenges, many modern forensic techniques, including convolutional neural networks, are showing increasing resilience to noise and image alterations. By training networks to recognize invariant features that remain consistent even under manipulation, these systems can improve the accuracy of camera identification. Furthermore, the use of large-scale datasets allows for better generalization of models to real-world conditions, making them more robust to unforeseen image quality degradations.

Deep learning models are increasingly vulnerable to adversarial attacks, where small, imperceptible changes to the input image can lead to misclassification. To address this, various defense mechanisms have been proposed to enhance the robustness of models against such attacks. These include adversarial training, where the model is trained on adversarially perturbed images to improve generalization, as well as defensive distillation, which involves training a secondary model to soften the predictions of the original model. Additionally, methods such as input preprocessing (e.g., denoising, feature squeezing) and model regularization techniques (e.g., dropout, weight decay) have been explored to reduce the model’s susceptibility to adversarial perturbations. For example, in the context of source camera identification, researchers have explored adversarial attacks on deep learning models for camera model recognition and have proposed defenses specifically tailored for camera forensics. For example, they include defense mechanisms that combine adversarial training with noise-based augmentation, achieving improved performance under attack scenarios. The results suggest that such hybrid defense strategies can help safeguard the integrity of camera source identification systems in real-world applications, where adversarial manipulation is a growing concern.

In conclusion, while statistical and sensor-based methods provide a solid foundation for camera identification, the continuous evolution of adversarial attack strategies demands constant adaptation of forensic algorithms. The ability to maintain high performance under altered conditions is vital for practical implementation in forensic applications. As such, there is a growing need for research that not only enhances the robustness of these techniques but also anticipates potential adversarial threats, ensuring that digital forensics can keep pace with evolving technologies.

## 9. Identification by Videos

Video-based identification utilizes unique characteristics within video data to trace recordings and link them to specific devices. The analyzed artifacts include sensor noise, compression patterns, and frame-level inconsistencies across videos recorded by the same device. This section presents techniques for extracting these identifying features from video, as well as their application and reliability in attributing video evidence to particular cameras.

The research presented in paper [122] explores the use of the PRNU pattern as a fingerprint for identifying individual cameras in digital footage, which may be particularly relevant in sensitive cases like child abuse and pornography. Utilizing a second-order method, the study extracts PRNU patterns from videos captured by ten different mobile phone cameras. The efficacy of SCI is evaluated by comparing the peak to correlation energy (PCE) of PRNU patterns from natural videos against reference flat field videos of each camera. This comparison is conducted on both original videos and those transmitted via WhatsApp on Android and iOS platforms. The findings indicate that most tested cameras yielded a high likelihood ratio for source identification. However, the study reveals that source camera identification is not feasible for videos transmitted through the iOS version of WhatsApp.

In the study [123], a method for identifying the source camera of videos, addressing a gap compared to image-based approaches is presented. The method utilizes camera-specific noise patterns extracted from video frames through an extended constrained convolutional layer that processes color inputs. Individual video frames are classified, and a majority vote is used to determine the source camera. Evaluated on the VISION dataset, the approach achieves an accuracy of up to 93.1% and demonstrates robustness against compression techniques used by platforms like WhatsApp and YouTube.

The inadequacies of existing image-based identification systems when applied to video frames are addressed in [124]. Recognizing that different forensic traces may be present in images and videos from the same camera, the authors propose a deep learning-based solution using a CNN to identify camera models from video content. The system operates by extracting small patches from temporally distributed frames, allowing for a comprehensive analysis of the video’s forensic information. A fusion system then combines the identification scores from the CNN to produce a more accurate overall result. The experimental results demonstrate the efficacy of this approach, achieving a 95.9% accuracy in video source camera identification.

The research [125] proposes an algorithm that identifies the source system of a video by analyzing features within the encoded stream. By utilizing distinctive characteristics in rate control strategies across video compression systems, the method extracts key features from the compressed stream and utilizes a support vector machine classifier to construct an identification framework. Experimental results demonstrate that the algorithm effectively distinguishes between video streams generated by various coding systems.

The paper [126] presents a robust method for video source identification, particularly suited to wireless video streams commonly used in security and surveillance. Traditional identification techniques struggle with wireless video distortions like blocking and blurring caused by packet loss. The proposed approach addresses this by utilizing wireless channel signatures and selective frame processing, which enhance both accuracy and processing speed. Extensive experiments show that this method achieves high identification accuracy, even with degraded video quality, and can detect wireless camera spoofing in real-time, offering an effective solution for validating video evidence and preventing piracy.

In [127], a method for identifying digital video cameras using the green-channel photo response non-uniformity (G-PRNU) as a camera fingerprint. By focusing on the green channel as known to be the noisiest after an exposure of 0.15 s, the G-PRNU is extracted, resized to 512×512 pixels using bilinear interpolation, and processed with a wavelet-based denoising filter. The fingerprint is then averaged across frames, and a 2D correlation coefficient is employed in detection. Tests on 290 video sequences from various devices demonstrate that G-PRNU outperforms traditional PRNU, showing the reliability of SCI.

The work [6] focuses on identifying source devices using videos through deep learning techniques. The study evaluates models with varying complexities and finds that, unlike traditional PRNU, noise-based methods need flat frames for camera pattern extraction. However, the proposed approach performs does not require such constraints. The testing across 28 devices from the VISION dataset reveals an accuracy of 72.75%, while performance on the QUFVD dataset achieves 71.75%. The paper also explores the impact of video content (e.g., flat, indoor, outdoor) and social media compression on classification results and highlights the method’s practical advantages, including runtime efficiency.

In [128], the impact of the lossy compression applied to the images used to estimate the fingerprint is considered. The paper investigates this issue both theoretically and experimentally, demonstrating a strong correlation between the theoretical predictions and experimental findings. The results highlight the significance of the study for video forensics, where higher compression rates are commonly encountered.

A summary of the SCI methods utilizing video footage is presented in Table 8.

### Discussion

Video-based identification extends the principles of image-based camera identification to the dynamic and complex environment of video data. Unlike static images, videos consist of temporal sequences of frames, providing additional information that can enhance identification accuracy.

One promising approach for video source identification is the use of sensor noise patterns, such as PRNU, as a fingerprint for the camera that captured the footage. In one study, the use of PRNU in videos was explored by analyzing the peak correlation energy (PCE) between PRNU patterns extracted from the video frames and reference flat-field videos. This method was tested on videos captured by mobile phone cameras and transmitted through messaging platforms such as WhatsApp. The results showed a high likelihood ratio for source identification when analyzing original videos, though challenges arose when videos were transmitted via WhatsApp, particularly on iOS devices, where the transmission process altered the identifying noise patterns, making it harder to trace the source camera.

The challenge of dealing with compression effects and transmission artifacts is significant when working with video-based identification methods, as these alterations can distort or mask the distinctive noise patterns in the video. Nevertheless, the study revealed that most cameras still produced sufficient PRNU patterns to allow for successful identification, particularly when the video remained unaltered by transmission platforms. Another innovative approach to video-based identification focuses on the extraction of camera-specific noise patterns directly from individual video frames. This technique involves convolutional layers that process the color information from each frame, extending the methodology typically used in still-image-based camera identification.

Overall, while video-based identification presents unique challenges due to the inherent complexity and dynamic nature of video data, techniques such as PRNU extraction and camera-specific noise pattern analysis show promising potential. The ability to identify source cameras from video evidence has important applications in forensic contexts, especially in cases where video authenticity is critical. The robustness of these methods against compression and transmission effects is an important area of ongoing research, as it influences their applicability in real-world scenarios.

## 10. Other Methods

In this section, we explore other methods that may be utilized to source camera identification.

In the paper by Jiang et al. [129], an approach called user camera identification (UCI), utilizing camera fingerprints to match social media accounts belonging to the same person is described. The main assumption is that multiple accounts will share similar camera fingerprint information. By focusing on this unique characteristic, UCI effectively addresses issues related to multiple cameras and reposting behaviors, providing a more reliable and robust solution for user identification across different social network platforms.

The paper proposed by Kozlov et al. [130], discusses the optimization methods for SCI. It considers the creation of noise signatures from homogeneous images and tests these signatures against images from three camera types. The study optimizes digital image filtering and identity metrics, selecting an optimal digital filter for smoothing images to extract noise signatures effectively. By comparing camera noise signatures using such filter and identity criteria, the reliability of identification is improved by over 60 times. Therefore, the results may be useful for application in image registration, processing, security, forensics, and big data analysis. The investigation of the efficacy of various wavelet transforms for SCI is presented in [131].

In the work by Wang et al. [132], the authors propose a method called the Envelope of Data Clustering Optimization (EDCO), which can identify camera models even if they are not included in the existing database. The experimental results demonstrate that EDCO effectively distinguishes between known and unknown source images, allowing for the accurate linking of known query images to their corresponding camera models. The proposed method outperforms state-of-the-art techniques in accurately identifying camera sources.

In the paper by Ding et al. [133], a domain knowledge-driven method for SCI, integrating hand-crafted and data-driven approaches to enhance feature extraction is proposed. The framework includes a preprocessing module that utilizes domain knowledge, a feature extractor, and a hierarchical multi-task learning procedure to provide information for a particular model identification. Evaluations across brands and models show that the method achieves an accuracy of 84.3% for cell phone identification, outperforming traditional camera identification methods.

In the paper proposed by Tsai et al. [134], extraction of features from images used for training data mining algorithms to determine the source cameras is considered. The study also compares cameras from different brands and similar models within the same manufacturer, revealing that feature-based approaches perform better in distinguishing between camera sources across brands.

The research by Zandi et al. [135] presents a method utilizing the weighted local binary pattern (WLBP) texture descriptor. The authors apply the WLBP operator specifically for camera classification to identify the imaging camera based on the two-dimensional histogram of Weber’s features and LBP. Experiments conducted on the Dresden Image Database demonstrate that the proposed method achieves a remarkable accuracy of 99.5% across nine different digital camera models. Even under JPEG compression with a quality factor of 70.0%, the method maintains an accuracy of 89.0%. These results indicate that the proposed approach not only offers high identification accuracy but also shows good robustness against compression artifacts compared to existing methods.

In the paper by Zheng et al. [136], a perceptual data-device hash that can both identify the source camera of an image and locate tampered regions, providing a non-repudiable attestation, is proposed. The hash is generated by projecting invariant image features into a physical unclonable function (PUF)-based Bernoulli random space, which is unique for each camera and triggered by the image acquisition timestamp. The proposed method achieves a high tamper detection rate of 95.4%, accurately locating tampered regions or geometric transformations. Additionally, the system identifies the SCI with 100.0% accuracy and is secure against PUF attacks.

The work by Deng et al. [137] proposes an approach to SCI by approximating the auto-white balance (AWB) algorithm used within cameras. This technique utilizes the consistency of the AWB process, which does not alter an image when applied repeatedly. This is the first known instance of utilizing AWB for camera identification. The experimental results demonstrate near-perfect accuracy in differentiating between various brands and models, as well as effectively distinguishing devices of the same model. The proposed method maintains stable performance even as the number of camera instances increases.

In the paper by Chen et al. [138], a framework for identifying the model of a camera by utilizing its demosaicing algorithm is proposed. Traditional approaches rely on parametric modeling of camera components, but this can be challenging due to the complexity and nonlinearity of the processing pipeline. The framework sidesteps these challenges, enabling highly accurate SCI. The experimental results demonstrate a high 99.2% accuracy in determining the correct make and model of the camera from the images.

The work by Geradts et al. [139] explores various methods to establish a connection between an image and a camera, including examining sensor defects, analyzing file formats, assessing noise patterns introduced by pixel arrays, and detecting manufacturer watermarking embedded in images.

A summary of the described SCI methods is presented in Table 9.

### Discussion

The diversity of methods presented in this section highlights the multifaceted nature of source camera identification and the growing range of techniques beyond traditional sensor noise analysis. These methods demonstrate that SCI is not limited to extracting physical sensor fingerprints but also encompasses broader strategies involving signal processing, feature extraction, machine learning, and the integration of domain knowledge.

In summary, these methods demonstrate the richness and evolution of SCI strategies. From low-level sensor artifacts to high-level camera processing characteristics and user behavior analytics, modern SCI methods are increasingly interdisciplinary, drawing from computer vision, signal processing, and cybersecurity. The field continues to mature, offering robust, scalable, and versatile solutions for identifying digital image sources under a wide range of real-world conditions.

## 11. Summary, Open Issues, and Future Work

The methods developed so far can be divided into several main categories. The most common technique is analyzing the matrix noise pattern PRNU, which is a unique “fingerprint” of a given sensor. In addition, methods are used based on the analysis of compression artifacts, as well as specific properties of image interpolation and filtration by signal processing algorithms in the camera. Recent years have also brought the development of methods based on deep learning, especially convolutional neural networks, which automatically learn the characteristics of different camera models.

Despite the high effectiveness of many of these methods, each has significant limitations. PRNU is susceptible to noise introduced by heavy lossy compression and aggressive image processing, making it challenging to identify a camera from heavily edited images. Methods based on EXIF metadata can be ineffective if the metadata is removed or falsified. Deep learning models, require large datasets for effective training and often have limited ability to generalize to unfamiliar lighting conditions or new camera models.

The most important challenges in digital camera identification concern both the resistance of methods to image modifications and the possibility of scaling the analyzed datasets. There is still a lack of methods that would be effective in low image quality conditions or after strong digital processing, e.g., adding filters, changing the contrast, or converting to black and white. Another problem is the possibility of falsifying the matrix “fingerprint” by deliberate modifications of PRNU noise, which can lead to false identifications. Additionally, the growing number of photographic devices and the evolution of image processing algorithms force the development of methods that not only identify a specific camera but are also able to adapt to new technologies.

Another issue is the importance of comparative evaluation across methods using a unified dataset. However, in the context of source camera identification, such direct comparison is often impractical due to significant differences in experimental setups, preprocessing pipelines, and target applications across various studies. Moreover, many methods rely on dataset-specific tuning or are designed for particular acquisition conditions, such as compression level, sensor type, or even specific device instances. As a result, creating a standardized benchmark remains a challenge in this domain. Nevertheless, we recognize the value of such an initiative and believe that developing common protocols for future evaluation could substantially benefit the research community.

Future research should focus on developing methods that are more resistant to image processing and enable identification based on fragmentary data. A key direction is the use of hybrid models that combine traditional approaches, such as PRNU analysis, with modern deep learning methods. In addition, it is worth developing algorithms capable of detecting manipulation and forgery, which will allow not only the identification of the camera but also the detection of attempts to interfere with digital evidence. An important area of research is also the automatic acquisition and analysis of large datasets in a computationally efficient manner, which will enable scaling of methods for the needs of real forensic systems.

## Figures and Tables

**Table 1:** Datasets for the source camera identification. The “I.D.” abbreviation stands for “image database”.

Ref.	Dataset Name	# of Images	# of Devices	Short Description
[8]	Dresden I.D.	14,500	73	images from digital cameras
[9]	MICHE-I	3700	92	iris biometric dataset, mobile devices
[10]	VISION	34,427	35	images and videos from digital cameras and mobile devices
[11]	Forchheim I.D.	23,000	27	images from mobile devices
[12]	UNIFI	5415	23	HDR images from mobile devices
[13]	IMAGINE	2816	67	images from digital cameras and mobile devices
[14]	QUFVD	—	20	6000 videos from digital cameras and mobile devices
[15]	SOCRatES	9700	103	images and videos from mobile devices
[16]	Daxing	43,400	90	images and 1400 videos from mobile devices

**Table 2:** Methods for source camera identification utilizing SPN analysis.

Reference	Short Description	Approach	Dataset	Accuracy
Mandelli et al. [28]	Fast identification using CNNs	Deep learning	VISION	97–99%
Qian et al. [27]	Three-stage extraction of SPN using deep learning	Deep learning	Dresden, VISION	96–99%
Zeng et al. [23]	SPN extraction near strong edges	Traditional	Dresden	96–98%
Kirchner et al. [26]	Using CNNs	Deep learning	Dresden, VISION	95–97%
Li et al. [20]	Principal component analysis	Traditional	Dresden	94–97%
Kang et al. [19]	Image edges analysis	Traditional	Dresden	92–96%
Kang et al. [18]	Analysis of artifacts, including JPEG compression	Traditional	Dresden	90–95%
Gupta et al. [25]	Analysis of low-frequency artifacts	Traditional	Dresden	91–94%
Soobhany et al. [22]	Image decomposition	Traditional	Custom	90–93%
Kulkarni et al. [24]	General sensor imperfections	Traditional	Dresden	89–92%
Satta [21]	Searching for high-frequency pixels	Traditional	Custom	85–90%

**Table 3:** Methods for the source camera identification utilizing the PRNU.

Reference	Short Description	Dataset	Accuracy
Gisolf et al. [33]	using a total variation noise removal algorithm	Dresden	up to 99%
Darvish et al. [35]	analysis of geometric operations applied to the images	UNIFI	up to 99%
Bernacki [41]		Dresden	up to 99%
Filler et al. [1]	identification from TIFF images	Dresden	65–98%
Kalka et al. [36]	identification of iris sensors	Different datasets	up to 96%
Tiwari et al. [38]	image edges and textures analysis	Dresden	up to 96%
Marra et al. [37]	clustering	Dresden	up to 96%
Ahmed et al. [50]	identification by CNN	Custom	up to 95%
Borole et al. [48]	analysis of the gray level co-occurrence matrix from PRNU	Dresden	up to 94%
Gupta et al. [34]	image edges and textures analysis	Dresden	up to 82%
Valsesia et al. [40]	compact representation of the PRNU	Dresden	n/a
Manisha et al. [42]	discussion about the limitations of the PRNU	Dresden	n/a
Lawgaly et al. [45]	analysis of additional non-unique components during the PRNU extraction	Dresden	n/a
Borole et al. [49]	using a fuzzy min-max neural network	Dresden	n/a
Balamurugan et al. [51]	three-stage extraction of the SPN	Dresden	n/a
Baar et al. [31]	clustering PRNU	Dresden	n/a
Tomioka et al. [39]	advanced analysis of the PRNU	Custom	n/a
Taspinar et al. [43]	using seam-carved images	Custom	n/a
Rodríguez-Santos et al. [44]	analysis by HDR images	Custom	n/a
Chen et al. [46]	image tampering detection	Custom	n/a
Yaqub et al. [47]	identification by cropped images	Flickr images	n/a
Fridrich [32]	analysis of sensor imperfections	n/a	n/a

**Table 4:** Source camera identification with deep learning.

Reference	Short Description	Dataset	Accuracy
Marra et al. [54]	iris sensor identification	Dresden	95–100%
Freire-Obregón et al. [56]	extracting features from images using high-pass filters	MICHE-I	80–100%
Bondi et al. [7]	identification directly from images	Dresden	56–99%
Marra et al. [65]	analysis of co-occurrence matrices of selected neighboring pixels	Dresden	85–99%
Tuama et al. [77]	analysis co-occurrence matrix and color filter array interpolation patterns	Dresden	up to 99%
Tuama et al. [30]	extracting features from images, using a high-pass filter	Dresden	93–98%
Chen et al. [57]	utilizing the low-level and high-level features	Dresden & Custom	up to 98%
Yang et al. [58]	identification by small-size images	Dresden	up to 98%
Yao et al. [52]	extracting features from images	Dresden	41–96%
Zhang et al. [70]	double compression analysis	Dresden	up to 96%
Elharrouss et al. [79]	pixel difference convolution and vision transformer	VISION, Daxing, SOCRatES, QUFVD	94, 84, 94, 92%
Tsai et al. [66]	software/hardware-related features of the images	Custom	up to 90%
Wang et al. [68]	software/hardware-related and statistical features of the images	SOCRatES, Dresden	n/a
Rafi et al. [53]	identification by post-processed images	Dresden	n/a
Liu et al. [59]	using particular patches for identification	Dresden	n/a
Bernacki et al. [62]	accelerating learning of CNNs	Dresden	n/a
Wang et al. [69]	identification based on a low number of images	Dresden	n/a
Wang et al. [72]	identification based on a low number of images	Dresden	n/a
Alhussainy et al. [76]	analysis co-occurrence matrix and color filter array interpolation patterns	Dresden	n/a
Debiasi et al. [55]	iris sensor identification	Custom	n/a
Lai et al. [74]	image acquisition and editing	Custom	n/a
Lu et al. [71]	using multi-scale feature fusion + transformer	n/a	n/a
Jaiswal et al. [73]	analysis of spatial features	n/a	n/a
Wang et al. [75]	identification by altered images	n/a	n/a
Bharathiraja et al. [78]	analysis of degrade images using deep learning	n/a	n/a

**Table 5:** Methods for the source camera identification utilizing aberrations.

Reference	Subject	Dataset	Accuracy
Van et al. [84]	lateral chromatic aberration	Custom	20–100%
San et al. [81]	intrinsic lens aberration and radial distortion	Custom	92%
Choi et al. [80]	lens radial distortion	Custom	81–91%
Bernacki [85]	utilizing lens vignetting and distortion	Dresden	52–84%
Yerushalmy et al. [82]	chromatic aberration for image forgery detection	Custom	n/a
Yu et al. [83]	chromatic aberration	Custom	n/a
Dirik et al. [86,87]	analysis the sensor dust	Custom	n/a

**Table 6:** Statistical methods for source camera identification.

Reference	Short Description	Dataset	Accuracy
Mieremet [91]	Mismatches of the PRNU	Dresden	95–97%
Xu et al. [94]	Utilizing texture features from color models	Dresden	96%
Thai et al. [90]	Analysis of gamma correction	Dresden	93–96%
Thai et al. [88]	Using discrete cosine transform	Dresden	92–95%
Agarwal et al. [92]	Identification using Haralick features, entropy, and image equality attributes	Dresden	92–94%
San et al. [89]	JPEG compression	Custom	90–93%

**Table 7:** Robustness and adversarial attacks for source camera identification techniques.

Reference	Short Description
Akshatha et al. [95]	image manipulating and adding noise
Bernacki [96]	altering image content by adding noise or removing the least significant bit
Chen et al. [97]	the impact of demosaicing
Samaras et al. [98]	pixel defects versus PRNU identification
Rosenfeld et al. [99]	denoising, recompression, out-of-camera demosaicing
Goljan et al. [100]	cropping and scaling images
Lin et al. [101]	adding noise to the images
Martin-Rodriguez et al. [102,103]	altering the image quality
Goodfellow et al. [104]	adding noise to the images
Moosavi-Dezfooli et al. [105]	adding noise to the images
Papernot et al. [106]	adding noise to the images
Cozzolino et al. [107]	injecting camera-specific traces into synthetic images
Chen et al. [108]	creating fake image
Sameer et al. [109]	anonymizing the image with the use of seam carving, fingerprint copying, and adaptive PRNU denoising
Yang/Li et al. [110,111]	physical interference to the lens
Wang et al. [112]	injecting camera’s fingerprint to another
Goljan et al. [113]	superimposing the fingerprint to the targeted image
Caldelli et al. [114]	
Li et al. [116]	
Quiring et al. [118]	
Barni et al. [119]	detecting if the PRNU was manipulated
Marra et al. [120]	manipulating the CNN used for SCI
Hsu et al. [121]	detecting image manipulations with camera response function

**Table 8:** Source camera identification by videos.

Reference	Short Description
Meij et al. [122]	identification by PRNU
Timmerman et al. [123]	utilizing camera-specific noise patterns extracted from video frames
Hosler et al. [124]	extracting small patches from temporally distributed frames
Su et al. [125]	analyzing the encoded stream
Chen et al. [126]	selective frame processing
Al et al. [127]	estimating the PRNU on a green channel
Bennabhaktula et al. [6]	PRNU + deep learning
Goljan et al. [128]	lossy compression

**Table 9:** The summary of different methods for source camera identification.

Reference	Short Description
Jiang et al. [129]	utilizing social media data
Kozlov et al. [130]	optimization of the SCI
Ding et al. [133]	integrating hand-crafted and data-driven approaches for feature extraction
Tsai et al. [134]	extraction of features from images
Zandi et al. [135]	utilizing the weighted local binary pattern texture descriptor
Zheng et al. [136]	perceptual data-device hash based on physical unclonable function (PUF)
Deng et al. [137]	auto-white balance
Chen et al. [138]	demosaicing algorithm
Geradts et al. [139]	sensor defects and noises analysis, watermarking detection

## Abbreviations

The following abbreviations are used in this manuscript:

ADF-Net	adaptive dual-branch fusion network
AWB	auto-white balance
BF	bacteria foraging
BISM	block image statistical measure
CRF	camera response function
CA	chromatic aberration
CCN	circular correlation norm
CFA	color filter array
CNN	convolutional neural networks
DNN	deep neural networks
DSLR	digital single-lens reflex
DCT	discrete cosine transform
DWT	discrete wavelet transform
DTCWT	dual-tree complex wavelet transform
EMD	empirical mode decomposition
EDCO	Envelope of Data Clustering Optimization
FM	feature modulator
FPN	fixed pattern noise
FRN	fusion residual networks
FMNN	fuzzy min-max neural network
GLRT	generalized likelihood ratio test
GAN	generative adversarial network
GCN	graph convolutional network
GLCM	gray-level co-occurrence matrix
HDR	high dynamic range
HOWE	high-order wavelet entropy
HF	high-frequency
HOWS	higher-order wavelet statistics
IQM	image quality measures
ISCI	individual source camera identification
JSD	Jensen–Shannon divergence
KLD	Kullback-Leibler divergence
LBP	local binary pattern
LCA	lateral chromatic aberration
LDA	linear discriminant analysis
LRT	likelihood ratio test
LF	low-frequency
MDM-CPS	multi-distance measures and coordinate pseudo-label selection
PCA	principal component analysis
PCE	peak to correlation energy
PCEP	Prototype Construction with Ensemble Projection
PDF	probability density function
PDN	pre-defense network
PRNU	photo response non-uniformity
PUF	physical unclonable function
ROC	receiver operating characteristic
RNFE	residual noise feature extractor
SCG	scaled conjugate gradient
SPN	sensor pattern noise
SlMoWT	single-level multi-orientation wavelet texture
SCI	source camera identification
SCMI	source camera model identification
SEA	spectrum-equalization algorithm
SE	squeeze-and-excitation
SVM	support vector machine
TM	texture measure
TTL	tri-transfer learning
UCI	user camera identification
ViT	vision transformer
WLBP	weighted local binary pattern
ZKP	zero-knowledge proof
